# Loss of CD44^dim^ Expression from Early Progenitor Cells Marks T-Cell Lineage Commitment in the Human Thymus

**DOI:** 10.3389/fimmu.2017.00032

**Published:** 2017-01-20

**Authors:** Kirsten Canté-Barrett, Rui D. Mendes, Yunlei Li, Eric Vroegindeweij, Karin Pike-Overzet, Tamara Wabeke, Anton W. Langerak, Rob Pieters, Frank J. T. Staal, Jules P. P. Meijerink

**Affiliations:** ^1^Princess Máxima Center for Pediatric Oncology, Utrecht, Netherlands; ^2^Department of Pediatric Oncology/Hematology, Erasmus Medical Center-Sophia Children’s Hospital, Rotterdam, Netherlands; ^3^Department of Immunohematology and Blood Transfusion, Leiden University Medical Center, Leiden, Netherlands; ^4^Department of Immunology, Erasmus Medical Center, Rotterdam, Netherlands

**Keywords:** human T-cell development, thymus, OP9-DL1, T-cell commitment, CD44, multi-lineage potential, gene expression

## Abstract

Human T-cell development is less well studied than its murine counterpart due to the lack of genetic tools and the difficulty of obtaining cells and tissues. Here, we report the transcriptional landscape of 11 immature, consecutive human T-cell developmental stages. The changes in gene expression of cultured stem cells on OP9-DL1 match those of *ex vivo* isolated murine and human thymocytes. These analyses led us to define evolutionary conserved gene signatures that represent pre- and post-αβ T-cell commitment stages. We found that loss of dim expression of CD44 marks human T-cell commitment in early CD7^+^CD5^+^CD45^dim^ cells, before the acquisition of CD1a surface expression. The CD44^−^CD1a^−^ post-committed thymocytes have initiated in frame T-cell receptor rearrangements that are accompanied by loss of capacity to differentiate toward myeloid, B- and NK-lineages, unlike uncommitted CD44^dim^CD1a^−^ thymocytes. Therefore, loss of CD44 represents a previously unrecognized human thymocyte stage that defines the earliest committed T-cell population in the thymus.

## Introduction

T-cell development in the thymus is a complex process that is accompanied by sequential transcriptional and epigenetic changes leading to T-cell lineage commitment while suppressing alternative cell fates (1, 2). T-cell development has been extensively studied in mice, while in humans it is less well defined due to limited availability of thymus material and inherent genetic diversity. Research has focused on mimicking the thymus environment using *in vitro* differentiation cultures starting with hematopoietic stem cells (HSCs) isolated from human cord blood or bone marrow. Historically, human–mouse hybrid fetal thymic organ cultures (FTOC) have been used to functionally define the various stages of human T-cell development (3). Later, OP9-DL1 cocultures proved more useful to study T-cell differentiation (4). Expression of NOTCH1 ligands including the Delta-like 1 ligand on bone marrow-derived stromal cells from *op/op* M-CSF deficient mice (5) induces NOTCH signaling in target cells of the hematopoietic lineage. NOTCH signaling promotes T-cell differentiation while inhibiting B-cell differentiation (6). The differentiation of HSCs in this coculture recapitulates human *in vivo* T-cell development as measured by the successive acquisition of CD7, CD5, CD1a, and CD4^+^CD8 surface markers (7, 8).

During development in the thymus, early T-cell precursors migrate within the cortex from which the positively selected CD4 and CD8 double positive (DP) thymocytes migrate to the medulla. As a result, developing thymocytes encounter specific signals at specific locations in the thymus (9). The OP9-DL1 coculture system lacks the typical thymus architecture that is required for proper T-cell development. It supports the development of early T-cell development until the DP stage. Yet, after prolonged culture few cells reach the CD4 and CD8 single-positive (SP) stage (10). These SP cells are functional and are generated independent of the presence of murine or human MHC class I expression on OP9-DL1 cells or the presence of dendritic cells; therefore, they have most likely been subject to positive selection based on interaction among T-cell precursors (11).

Early T-cell progenitors (ETPs) are uncommitted, multipotent thymocytes that retain the ability to develop into hematopoietic cells other than the T-cell lineage including NK-cells, B-cells, and cells of the myeloid and erythroid lineages (12–15). Fully committed thymocytes have lost multipotency and have undergone RAG1/2-mediated T-cell receptor (TCR) alpha and beta chain rearrangements. Based on earlier studies, human CD4/8 double-negative (DN) thymocytes express CD1a at the proliferation stage when committed to the T-cell fate (16). The human HSC marker CD34 is gradually lost throughout development. However, CD34 was demonstrated as a poor marker for “stemness” of uncommitted thymocytes as it remains expressed (albeit dimly) on most CD1a^+^ T-cell committed thymocytes (13, 16, 17). Although upregulation of CD1a is generally used to define human T-lineage commitment (13), the human DN thymocyte maturation stages and the exact T-cell commitment point have not yet been clearly defined.

In this study, we generated gene expression profiles of early T-cells representing sequential human thymocyte differentiation stages. These have been derived from umbilical cord blood (UCB) stem/progenitor cells that have full multi-lineage differentiation potential and that were cultured on OP9-DL1 stromal cells. Comparisons of these *in vitro*-derived signatures with the gene signatures from *in vivo* normal murine and human early T-cell development stages in the thymus reveal strong conservation of pre- and post-T-cell commitment transcriptional profiles. From these analyses, we found that loss of human *CD44* expression and loss of CD44 at the surface membrane of early DN thymocytes marks T-cell commitment. Commitment is validated by the initiation of *TCRB* recombinations and loss of potential for alternative cell fate decisions.

## Materials and Methods

### Human UCB and Thymus Samples

Human UCB samples were obtained from consenting mothers after delivery at local hospitals. Mononuclear cells (MNCs) were isolated by Ficoll-Paque density centrifugation, washed, and frozen in 10% dimethyl sulfoxide and 90% fetal bovine serum (FBS) for later use. Thymi were obtained as surgical tissue discards from infants 2–9 months of age undergoing cardiac surgery at Erasmus MC Rotterdam, after informed consent from the parents or legal guardians. The children did not have immunological abnormalities. Thymocytes were isolated by cutting the thymic lobes into small pieces and squeezing them through a metal mesh and stored at −80°C until further analyses. Informed consents were in accordance with the Institutional Review Board of the Erasmus MC Rotterdam and in accordance with the Declaration of Helsinki.

### Isolation of CD34^+^ Cells from UCB

Frozen MNCs were thawed, washed, and labeled with MicroBeads conjugated to the monoclonal mouse anti-human CD34 antibody according to the manufacturer’s procedure (Miltenyi Biotec). The magnetic separation was performed twice using two MACS Columns (Miltenyi Biotec), consistently reaching a CD34^+^ purity of approximately 95%.

### Antibodies

Antibodies (with clone identification) used for flow cytometry: CD1a (HI149), CD3 (UCHT1), CD7 (M-T701), CD10 (HI10a), CD11b (ICRF44), CD13 (WM15), CD14 (MΦP9), CD20 (L27), CD22 (HIB22), CD33 (WM53), CD34 (8G12) (BD Biosciences), CD3 (BW264/56), CD4 (VIT4), CD5 (UCHT2), CD8 (BW135/80), CD19 (LT19), CD33 (AC104.3E3), CD34 (AC136), CD44 (DB105), CD45 (5B1), CD56 (REA196), CD94 (REA113), CD123 (AC145) (Miltenyi Biotec).

### OP9-DL1 Cocultures

OP9-DL1 cocultures were performed according to the original protocol (18). Briefly, CD34^+^ human HSC isolated from UCB was plated into 100 or 145 mm culture dishes (Greiner Bio-One B.V.), which were seeded with a monolayer of OP9-DL1 cells beforehand. The α-minimal essential medium (Life Technologies) used for culture was supplemented with 20% FBS (Integro B.V.) plus 10 U/mL penicillin, 10 µg/mL of streptomycin, and 0.025 µg/mL fungizone (PSF) (Life Technologies). Recombinant human stem cell factor (SCF) (10 ng/mL) (R&D systems), FLT3L (5 ng/mL) (Miltenyi Biotec), and interleukin-7 (IL7) (2 ng/mL) (Miltenyi Biotec) were added at the initiation of coculture and every 2–3 days during transfer of HSC-derived cells onto new OP9-DL1 plated cells. Cocultures were separated by vigorous pipetting and put through a 40-µm filter to reduce stromal cell line aggregates and eliminate contaminating OP9-DL1 cells.

Multi-lineage differentiation experiments were performed with the following modifications: recombinant human SCF (10 ng/mL), FLT3L (5 ng/mL), IL7 (5 ng/mL), and IL15 (10 ng/mL) were added every 3–4 days to half of the medium that was refreshed without disturbing the cells. A continuous 10-day OP9-coculture was initiated with 1,000 OP9 (-DL1, -GFP, or 1:1 mix) cells/well plated in 96-well plates 1 day before adding 4,000/well of the sorted thymocytes.

OP9-GFP and OP9-DL1 cell lines were kindly given to us by Dr. Zúñiga-Pflücker; the identity of the cell lines was confirmed by DNA fingerprinting, and cells were regularly tested for mycoplasma contamination.

### TCRB Rearrangements

In-frame Dβ–Jβ and Vβ–Jβ gene rearrangements were determined using the BIOMED-2 multiplex PCR and visualized using GeneScan (19). Primers for the Jβ1 cluster were hexachloro-6-carboxy-fluorescein (HEX) labeled (blue traces), and primers for the Jβ2 cluster were 6-carboxy-fluorescein (FAM) labeled (green traces).

### Cell Sorting and RNA Preparation

Throughout the differentiation of HSC toward the T-cell lineage, we sorted T-cell fractions at various time points (days 0, 7, 12, 18, and 27) based on the expression of cell surface markers including CD45, CD34, CD7, CD5, CD1a, CD4, and CD8. Cell sorting purity was >98% and performed using the FACSAria II (BD Biosciences), flow cytometry was performed using the MACS Quant (Miltenyi Biotec), and data analysis was performed using the FlowJo software (Tree Star Inc.). Sorted populations (range: 6.8 × 10^4^–2.68 × 10^6^ cells/population, median: 2.62 × 10^5^ cells) were collected, washed twice in PBS, lysed in buffer RLT (Qiagen) plus 1/100 β-mercaptoethanol, and stored at −80°C. RNA was isolated using RNeasy Micro Kit according to the manufacturer’s protocol (Qiagen).

### Affymetrix Gene Expression Arrays

The quality control, RNA labeling, hybridization, and data extraction were performed at ServiceXS B.V. (Leiden, the Netherlands). RNA concentration was measured using the Nanodrop ND-1000 spectrophotometer (Nanodrop Technologies). The RNA quality and integrity was determined using Lab-on-Chip analysis on the Agilent 2100 Bioanalyzer (Agilent Technologies, Inc.) and/or on the Shimadzu MultiNA RNA analysis chips (Shimadzu Corporation). Biotinylated cRNA was prepared using the Affymetrix 3′ IVT Express Kit (Affymetrix) according to the manufacturer’s specifications with an input of 100 ng total RNA. The quality of the cRNA was assessed using the Shimadzu MultiNA in order to confirm if the average fragment size was according to Affymetrix’ specifications. Per sample, 7.5 µg cRNA of the obtained biotinylated cRNA samples was fragmented and hybridized in a final concentration of 0.0375 µg/µL on the Affymetrix HT HG U133+ PM (Affymetrix). After an automated process of washing and staining by the GeneTitan machine (Affymetrix) using the Affymetrix HWS Kit for GeneTitan (part nr. 901530), absolute values of expression were calculated from the scanned array using the Affymetrix Command Console v3.2 software.

The data discussed in this publication have been deposited in NCBI’s Gene Expression Omnibus (20) and are accessible through GEO Series accession number GSE79379.^1^

### Taqman Array Microfluidic Cards

Reverse-transcriptase (RT) reactions (Promega) were performed to convert mRNA into cDNA. The real-time PCR (qPCR) reactions were performed by Taqman Custom arrays that were pre-designed according to the manufacturer instructions (Applied Biosystems). TaqMan gene expression assay targets were based on selected primers that were pre-loaded into each of the wells of a 384-well Taqman array card. TaqMan Gene Expression Assays consist of a pair of unlabeled PCR primers and a TaqMan probe with a FAM or VIC dye label on the 5′ end and minor groove binder and non-fluorescent quencher on the 3′ end. For the reaction, cDNA samples were diluted and mixed in 1:1 ratio with Taqman Fast Universal PCR Master Mix (Applied Biosystems). Relative levels of gene expression are determined from the fluorescence data generated during PCR using the 7900HT Fast Real-Time PCR System Relative Quantitation software (Applied Biosystems).

### Bioinformatics

Affymetrix gene expression CEL files were processed using Partek^®^ Genomics Suite^®^ 6.6. Robust Multi-array Average (21) was applied prior to further analysis. In brief, the intensity levels were quantile normalized after background correction. Probeset-level expression values were summarized by median polish approach and eventually log_2_ transformed. Probesets were filtered for high variance (top 5%) and log_2_ expression values (>8), which resulted in 2,179 probesets. Using the annotation file of the array, these probesets were then summarized into 1,387 genes by taking the median of all probesets across a gene. Principal component analysis (PCA) and hierarchical clustering were done using Partek^®^ Genomics Suite^®^ 6.6. Log_2_ expression values were standardized into *z*-scores prior to the clustering analysis. Pearson dissimilarity and Euclidean distance were used as distance metric. Gene set enrichment analysis (GSEA) (22) was performed using GSEA software downloaded from Broad Institute.^2^ GSEA is broadly used to analyze genome-wide expression profiles from samples belonging to two classes, in order to determine whether an *a priori* defined set of genes is correlated with the phenotypic class distinction. The method derives its power by focusing on gene sets instead of single genes. In this study, we took an unbiased approach at defining the gene sets using an unsupervised clustering method. For the OP9-DL1 *in vitro* dataset, the GSEA input gene list consisted of 1,387 genes that were grouped into the 16 gene signatures identified by the clustering analysis. The T-cell populations were denoted as early or late T-cell program according to their gene expression profile similarities during T-cell differentiation. Gene sets with false discovery rate (FDR) *q* ≤ 0.25 were considered significantly correlated to the class distinction (22). The murine *in vivo* microarray data was obtained through access to the Immunological Genome Project data, GEO accession code: GSE15907 (23). For analysis of this *in vivo* dataset, the GSEA input gene list consisted of 547 genes enclosed in the 5 signatures that form the T-cell development gene expression signature. The human *in vivo* microarray data were obtained from Dik et al. (16), MIAME accession no. E-MEXP-337. Hierarchical clustering on the human thymocyte populations was performed using 399 out of 547 genes from the T-cell development gene signature. The remaining 148 genes could not profiled in the human dataset because the U133A array used in Ref. (16) contains fewer probesets than the U133 plus 2.0 array used by us.

Relative levels of gene expression data generated by the Taqman array microfluidic cards were processed using Partek^®^ Genomics Suite^®^ 6.6. The sorted *in vitro* populations (A–F) were subject to ANOVA analysis to remove day and pool effects prior to the hierarchical clustering. Standardized *z*-scores of the expression values in 47 selected T-cell development genes were clustered using Pearson dissimilarity measure and average linkage. These 47 genes are a subset of the 1,387 genes with high variation and expression levels in our *in vitro* T-cell differentiation dataset. The same genes and clustering method were used for the human thymocyte populations (I–III) dataset. For this dataset, the expression values obtained by the microfluidic cards were subject to quantile normalization before further analysis.

## Results

### Consecutive Stages of Human *In Vitro* T-Cell Differentiation Represent Two Major Gene Signatures

**Figure 1:**
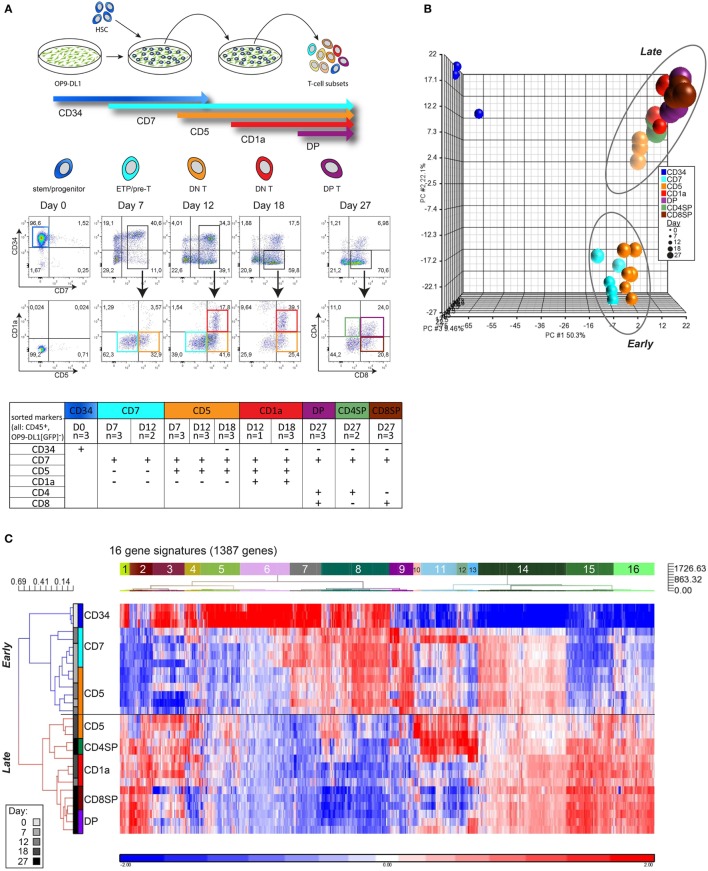

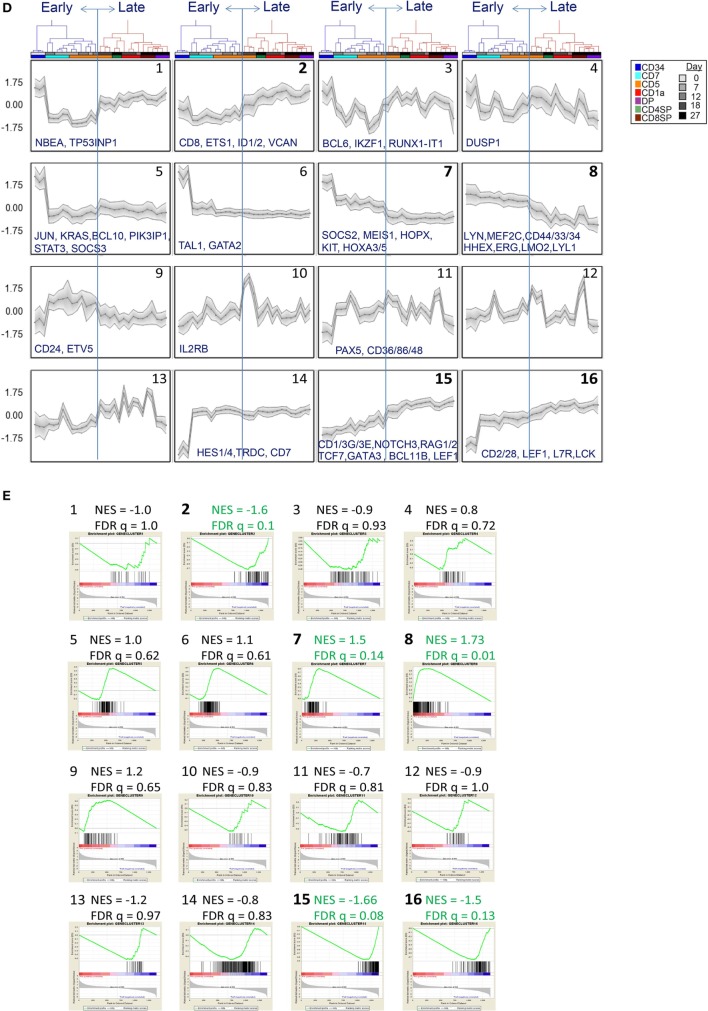
**Consecutive stages of human early *in vitro* T-cell differentiation represent two major gene signatures**. **(A)** Schematic representation of the OP9-DL1 coculture with the sorting strategy of consecutive T-cell differentiation stages. The table displays the sorted populations (and the replicates, from a total of five cocultures) based on surface markers (CD45, CD34, CD7, CD5, CD1a, CD4, CD8) and number of days in coculture. All populations were also gated for CD45^+^GFP^−^ to exclude OP9-DL1 cells that are GFP positive. **(B)** Principal component analysis of 29 samples based on the 2,179 probesets with high variance (top 5%) and log_2_ expression values (>8). These probesets were summarized into 1,387 genes by taking the median of all probesets across a gene. UCB-derived CD34^+^ stem cells (dark blue, upper left corner), an early, and a late population can be distinguished. **(C)** Hierarchical clustering analysis using 1,387 genes (see Table S1 in Supplementary Material). Pearson dissimilarity measure and average linkage were applied to cluster the samples, and Euclidean distance and Ward’s method were applied to define 16 distinct gene signatures with similar expression patterns. **(D)** Average expression pattern of genes in the 16 gene signatures; some example genes are displayed. *z*-scores are on the *y*-axis and 29 populations on the *x*-axis, ordered according to Figure 1C. The gray area represents 1SD. **(E)** Gene set enrichment analysis on the *in vitro* T-cell differentiation dataset to select gene signatures that are significantly enriched in the early or late T-cell program (marked in green and bold). The Normalized Enrichment Score (NES) and false discovery rate (FDR) *q*-value for each gene signature are given on top of each sub-figure. Populations belonging to the early/late T-cell program were determined according to Figure 1C. Gene sets with FDR *q* ≤ 0.25 were considered significantly correlated to the class distinction (22).

We isolated CD34^+^ HSCs from human UCB and cultured these cells on OP9-DL1 stromal cells to induce T-cell differentiation. At various time points during 27-day cocultures (performed in biological triplicate), seven distinct T-cell populations were sorted as progeny from pooled CD34^+^ HSCs from multiple cord blood donors (Figure 1A). Microarray gene expression analysis was performed on 26 OP9-DL1-generated T-cell populations collected from five independent cocultures and three CD34^+^ HSC starting pools. PCA revealed three distinct clusters (Figure S1A in Supplementary Material). After filtering for probes with high variance (top 5%) and high log_2_ expression (>8), the expression profile was trimmed to 1,387 genes (2,179 probesets) that robustly separate the three PCA clusters (Figure 1B; Table S1 in Supplementary Material). One cluster (upper left corner) represents the gene expression profile of CD34^+^ cells. The other two developmental clusters characterize “early” T-cell progenitor (ETP) populations (including the CD7^+^ and part of the CD7^+^CD5^+^ sorted populations) and “late” T-cell differentiation populations (part of the CD7^+^CD5^+^ population and CD7^+^CD5^+^CD1a^+^, CD4/8 DP, and SP sorted populations). The “early” and “late” clusters represent a major change in the transcriptional program (Figures 1B,C). Of note, the CD7^+^CD5^+^ population (in orange) is divided over both clusters (Figure 1B); those fractions that were sorted on days 7 and 12 of the coculture cluster with early CD7^+^ populations, whereas those sorted on day 18 fall in the “late” cluster. Therefore, this CD7^+^CD5^+^CD1a^−^ population is not one homogeneous population but reflect two distinct differentiation stages that dramatically differ in their transcriptional programs.

The 1,387 genes of our expression profile can be further differentiated into 16 different gene signatures of co-expressed genes (Figures 1C,D; Table S1 in Supplementary Material). Based on GSEA, we identified five signatures (#2, #7, #8, #15, and #16) that are most differentially expressed between the early and late T-cell differentiation populations (Figure 1E). Signatures #7 and #8 are enriched for genes that are highly expressed in the early T-cell progenitor stages but downregulated at later T-cell differentiation stages. Signatures #2, #15, and #16 are enriched for genes that become expressed at later T-cell differentiation stages. These five gene signatures—comprising 547 genes in total—faithfully distinguish the early and late T-cell differentiation populations (Figures S1B,C and Table S1 in Supplementary Material). Early T-cell differentiation signatures #7 and #8 include transcription (co)factors that are important for HSC/ETP maintenance and/or lineage determination, such as *MEIS1, LMO2, MEF2C, LYL1*, and *HHEX* (24–26). Genes encoding transcription factors that are responsible for T-cell identity (including *GATA3, TCF7, BCL11B*, Figure S2 in Supplementary Material) and TCR rearrangements (*RAG1/2*) are present in late signatures #2, #15, or #16. Therefore, these five signatures contain essential genes that are associated with transcriptional programs required for stem/progenitor identity, T-cell specification, and lineage commitment.

### The *In Vitro* Gene Expression Signatures Recapitulate *In Vivo* Signatures of Pre- and Post-T-Cell Committed Thymocytes

**Figure 2:**
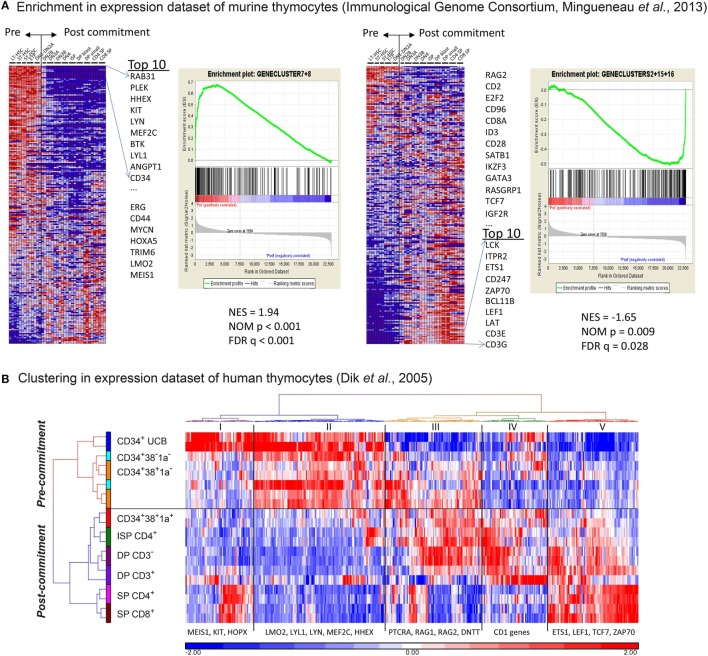
**The *in vitro* gene expression signatures recapitulate *in vivo* signatures of pre- and post-T-cell committed thymocytes**. **(A)** Gene set enrichment analysis of our *in vitro* gene signature on the gene expression dataset of consecutive T-cell development populations from murine thymi (see Table S2 in Supplementary Material). The first and second sub-figures from the left are the analysis results using gene signatures #7 and #8. The Normalized Enrichment Score (NES), Nominal *p*-value, and false discovery rate *q*-value indicate that these two gene signatures correlate with the pre-commitment stages. Also shown in the first sub-figure is the list of top 10 input genes with the highest signal-to-noise ratios in this dataset. The third and fourth sub-figures are the same analysis for gene signatures #2, #15, and #16, which correlate with the post-commitment stages. **(B)** Hierarchical clustering (Euclidean distance and Ward’s method) on human thymocyte populations was performed using 399 out of the 547 *in vitro* pre-/post-commitment signature genes (see Table S3 in Supplementary Material); the remaining 148 genes were not profiled because they were not on the HG-U133A array used in the dataset in Ref. (16). The dendrogram on the left shows the clustering of different sorted human thymocytes, separating the pre- and post-committed populations.

We then compared our gene expression signatures to those of murine and human *ex vivo* sorted thymocyte subsets (16, 23, 25). We performed GSEA to determine the enrichment of our five gene signatures in the dataset of consecutive T-cell development populations from murine thymi, generated by the Immunological Genome Consortium (23). As a result, we demonstrate that our “early” signatures #7 and #8 are significantly enriched for genes that are typically expressed in uncommitted murine subsets ranging from long-term HSCs to DN1–DN2a stages (Figure 2A; FDR-corrected *p*-value is *q* < 0.001). Known pre-commitment genes such as *HHEX, KIT, MEF2C, LYL1* are among the top 10 genes in our early signatures (Figure 2A: left panel; Table S2 in Supplementary Material). Our “late” T-cell development gene signatures #2, #15, and #16 are significantly enriched for genes expressed in post-committed murine thymocyte subsets (DN2b and later subsets) and include *BCL11B, TCF7, LEF1, GATA3* and various molecules associated with TCR signaling (FDR *q* = 0.028, Figure 2A: right panel; Table S2 in Supplementary Material). We then compared our gene signatures to gene expression data of human *ex vivo* sorted thymocyte populations (16). Interestingly, our gene signatures that discriminate pre- and post-commitment also divide the *ex vivo* sorted human thymocytes into uncommitted (the CD1a^−^ populations) and T-cell committed populations (CD1a^+^ and beyond; Figure 2B; Table S3 in Supplementary Material). These results show that the pre- and post-commitment programs in murine and human *in vivo* T-cell development remain highly conserved in T-cells that are generated from HSCs cultured on OP9-DL1 stromal support.

### Loss of CD44 Surface Expression Marks T-Cell Commitment in Early Human Thymocytes

**Figure 3:**
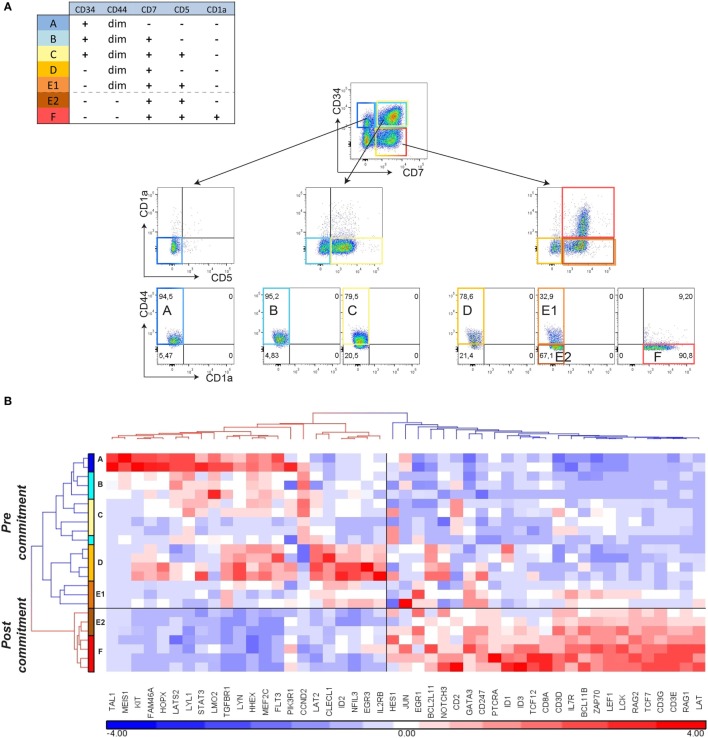
**Loss of CD44 surface expression marks T-cell commitment *in vitro***. **(A)** Sorting strategy for isolating T-cell differentiation stages A–F from duplicate (*n* = 2) 12- and 18-day cocultures started with pooled CD34^+^ hematopoietic stem cells, based on surface markers CD45, CD34, CD7, CD5, CD1a, and CD44. **(B)** Hierarchical clustering (Pearson average) of sorted populations A–F using 47 selected T-cell development genes. Expression values were obtained by Taqman array microfluidic cards, followed by ANOVA to remove day and pool effects.

Further examination of the genes that are expressed in the *ex vivo* CD34^+^CD1a^−^ human thymocyte populations (16) revealed expression of certain post-commitment genes [including *PTCRA, RAG1, RAG2*, and *DNTT* (known as *TDT*)]. This suggests that thymocytes prior to their acquisition of CD1a surface expression have initiated *TCRB* rearrangements and are already committed to the αβ T-lineage (Figure 2B; Table S3 in Supplementary Material). From our gene signatures, we searched for surface antigens that are differentially expressed between pre- and post-commitment stages. We observed that expression of *CD44, CD33, CD63, CD69*, and *CD300LF* were markedly reduced before the appearance of CD1a on the cell surface (Figure 1D; Figure S2 in Supplementary Material). As the DN2a–DN2b T-cell commitment point in murine T-cell development is associated with an initial reduction of CD44 expression (2, 23), we investigated whether loss of CD44^dim^ surface expression marks human T-cell commitment. CD44 surface expression on differentiating T-cells in the OP9-DL1 system is lost from CD5^+^ T-cells before these acquire CD1a expression (Figure 3A). We sorted subsets at days 12 and 18 from two independent OP9-DL1 cocultures with the inclusion of CD44 as an additional sorting marker and were able to purify CD5^+^CD44^dim^CD1a^−^ (E1), CD5^+^CD44^−^CD1a^−^ (E2), and CD5^+^CD44^−^CD1a^+^ (F) populations (Figure 3A). Microfluidic cards-based RT-qPCR analysis of 47 pre- or post-commitment genes strongly supported a pre-commitment profile in sorted populations A through E1, and a T-lineage commitment profile in populations E2 and F (Figure 3B). Therefore, T-cell commitment of differentiating T-cells cultured on OP9-DL1 is associated with loss of CD44^dim^ expression before the acquisition of CD1a surface expression.

**Figure 4:**
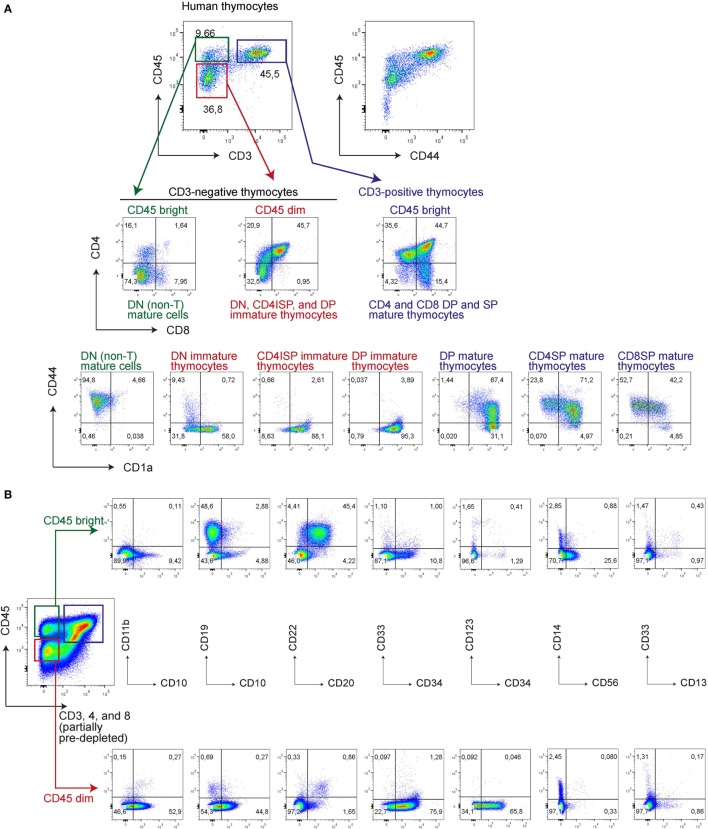
**CD45^bright^ are mature and CD45^dim^ are immature human thymocytes, correlating with the intensity of CD44 expression**. Human thymocytes were thawed and stained directly **(A)** or after CD3, CD4, and CD8 (partial) depletion of ~95% of all thymocytes **(B)** with various antibodies as indicated.

Next, we determined whether loss of CD44 during *in vitro* T-cell differentiation from UCB-derived CD34^+^ stem cells parallels T-cell commitment during *in vivo* human T-cell development. For this, we first assessed CD44 expression in human thymocytes in relation to surface expression levels of CD45, CD3, and other T-cell markers (Figure 4). CD44 expression on early, uncommitted thymocytes is dim in comparison to the bright CD44 expression on mature T-cells (27), and roughly correlates with CD45 expression levels (Figure 4). CD45^dim^ thymocytes are CD44^dim^ or CD44^−^ and predominantly consist of developmentally early thymocyte fractions that include CD4/8 DN, CD4 immature single positive (ISP), and some CD3^−^ DP cells. The DN thymocytes contain the CD44^dim^, CD44^−^CD1a^−^, and CD44^−^CD1a^+^ populations, whereas the later CD4ISP and immature CD3^−^ DP cells are all CD44^−^CD1a^+^ (Figure 4). In contrast CD45^bright^ thymocytes are CD44^bright^ and mostly consist of CD3^+^ DP, CD4 SP, and CD8 SP thymocyte populations. The human thymus also contain very low numbers of mature B-, myeloid, and NK-cells (17), that are CD3/4/8 triple-negative but CD45^bright^CD44^bright^, and CD1a^−^ (Figure 4).

**Figure 5:**
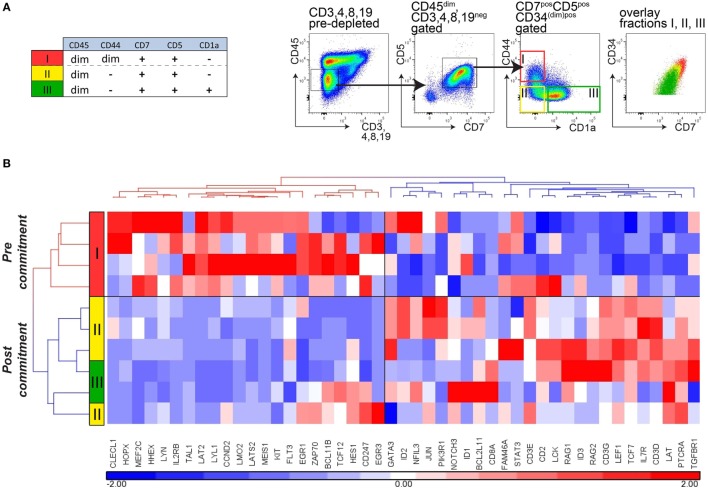
**Loss of CD44 surface expression marks T-cell commitment in early human thymocytes**. **(A)** Sorting strategy for isolating T-cell developmental stages I–III from four independent donors (*n* = 4), based on CD45, CD7, CD5, CD1a, and CD44 after pre-depletion of CD3-, CD4-, CD8-, and CD19-expressing thymocytes. **(B)** Hierarchical clustering (Pearson average) of sorted populations I–III using 47 selected T-cell development genes. Expression values were obtained by Taqman array microfluidic cards, followed by quantile normalization.

We then sorted immature (CD34^+^)CD7^+^CD5^+^ thymocytes from CD45^dim^ cells (lacking CD45^bright^CD44^bright^ cells) from thymi of four independent and immunologically healthy donors. Following depletion of CD3, CD4, CD8, and CD19 cells, CD7^+^CD5^+^CD45^dim^ immature thymocytes were further sorted into three populations: CD44^dim^CD1a^−^ cells (population I), CD44^−^CD1a^−^ cells (population II), and CD44^−^CD1a^+^ cells (population III; Figure 5A). These *ex vivo*-sorted populations I–III are equivalent to the *in vitro* populations E1, E2, and F (Figure 3A). Hierarchical cluster analysis of the gene expression levels of the panel of 47 pre- and post-commitment genes identifies population I (CD44^dim^) as uncommitted thymocytes, whereas populations II and III CD44^−^ thymocytes have committed to the T-cell lineage (Figure 5B). Therefore, loss of CD44^dim^ expression marks the transition to human T-cell commitment *in vivo*, consistent with our *in vitro* data. Of note, expression of the stem-cell marker CD34 gradually decreased in populations I–III but was still present on a fraction of CD1a^+^ thymocytes (population III, Figure 5A), in line with previous observations (13, 16). Therefore, loss of CD34 surface expression does not mark T-cell commitment.

### Loss of CD44 Functionally Marks Human T-Cell Commitment

**Figure 6:**
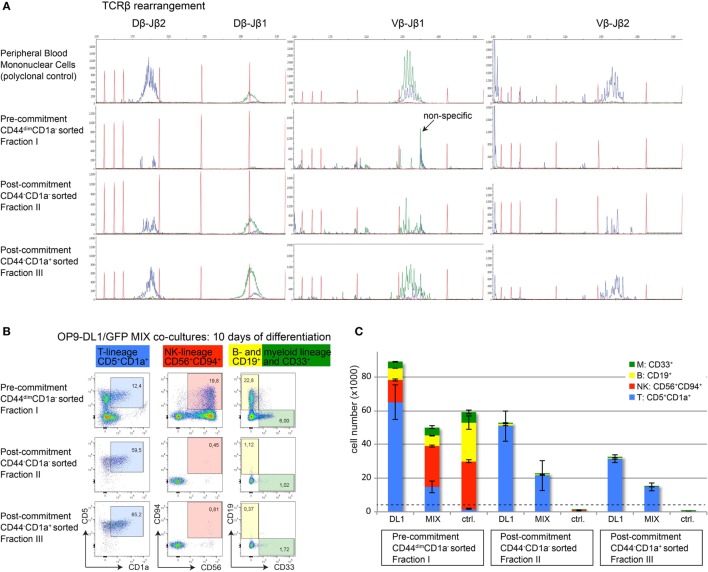
**Loss of CD44 functionally marks human T-cell commitment**. **(A)** GeneScan visualization of in-frame Dβ–Jβ (left column) and Vβ–Jβ (middle and right columns) gene rearrangements, determined using the BIOMED-2 multiplex PCR (19). One representative example of three independent donors is shown. Healthy peripheral blood mononuclear cells were used as a positive, polyclonal control (top row); populations I–III are indicated (rows 2–4). Primers for the Jβ1 cluster were hexachloro-6-carboxy-fluorescein-labeled (blue traces), and primers for the Jβ2 cluster were 6-carboxy-fluorescein (FAM)-labeled (green traces). Red traces: internal size markers. **(B)** Flow cytometry analysis of sorted human thymocyte populations I–III after 10 days of coculture on OP9-DL1:GFP mixed cells. Differentiation into various hematopoietic lineages is defined as follows: T-lineage (blue): CD5^+^CD1a^+^, NK-lineage (red): CD56^+^CD94^+^, B-lineage (yellow): CD19^+^, myeloid lineage (green): CD33^+^. During differentiation, all populations remain CD45^+^ and CD7^+^. **(C)** Absolute cell numbers ± SD of each differentiated and proliferated hematopoietic lineage from a representative of three experiments, after 10 days of coculture on a layer of OP9-DL1, a 1:1 mix, or OP9-GFP (ctrl.) cells. The number of cells from populations I–III used to initiate the cocultures on day 0 was 4,000 cells/well, indicated by the dashed horizontal line.

αβ T-cell committed thymocytes have initiated or completed *TCRB* gene rearrangements. To further validate T-cell commitment in sorted CD44^−^CD1a^−^ (population II) and CD44^−^CD1a^+^ (population III) human thymocytes, we analyzed Dβ–Jβ and Vβ–Jβ recombination events using GeneScan analysis (19). Analysis of sorted CD44^dim^ uncommitted thymocytes (population I) did not reveal detectable rearrangements (Figure 6A). In contrast, incomplete Dβ–Jβ and Vβ–Jβ recombination events were identified in the earliest population of post-committed CD44^−^CD1a^−^ thymocytes (II) with a more complete spectrum of full recombinations present in CD44^−^CD1a^+^ thymocytes (III) (Figure 6A).

To provide functional evidence that loss of CD44^dim^ marks T-cell commitment, we investigated the intrinsic potential of alternative cell fate decisions in the three sorted human thymocyte populations I–III. For this, thymocytes from populations I–III were cultured on OP9-DL1 stromal cells, on OP9-GFP control cells that do not support T-cell differentiation or on a 1:1 mixture of OP9-DL1/OP9-GFP stromal cells. These cultures were performed in the presence of cytokines that support differentiation into various hematopoietic lineages. After seven days of coculture, microscopic inspection already revealed the inability of committed thymocyte populations II and III to proliferate in cocultures with OP9-GFP stromal cells (Figure S3 in Supplementary Material). After 10 days of coculture under various stromal support conditions, we determined the total numbers of T-, NK-, B-, and myeloid cells based on lineage markers CD5 and CD1a (T), CD56 and CD94 (NK), CD19 (B), and CD33 (myeloid) (Figures 6B,C). The uncommitted thymocytes (population I) proliferated well and differentiated into cells of all four lineages on OP9-DL1 support. This uncommitted population efficiently gave rise to B-, myeloid, and NK-cells—but not T-cells—when cultured on OP9 control cells. In contrast, committed thymocyte populations II and III gave rise to CD5^+^CD1a^+^ T-cells only, indicating that these populations have lost the potential to differentiate into alternative cell fates (Figure 6C). The fact that both populations II (CD44^−^CD1a^+^) and III (CD44^−^CD1a^−^) have fully adopted the T-cell fate illustrates that loss of CD44—rather than the acquisition of CD1a—is the first surface marker that identifies post-committed human thymocytes.

## Discussion

The early stages of human T-cell development have been investigated (28) without or with gene expression profiling (16), using FTOC cultures (3, 29, 30), and more recently cocultures on OP9-DL1 (11). During the last decade, the OP9-DL1 *in vitro* coculture system has been used widely to study T-cell development (31). The use of human hematopoietic progenitors in this culture system offers a relatively easy and efficient tool to translate studies from *in vivo* mouse models to the human situation (11). Sequential acquisition of T-cell specific surface markers recapitulates T-cell development from bone marrow or cord blood hematopoietic progenitors to the DP stage (8). Here, we have sorted subsets at different stages during early T-cell differentiation and demonstrated conservation of early T-cell transcriptional profiles including pre- and post-T-cell commitment programs compared to *in vivo* mouse and human T-cell development. We identified loss of CD44^dim^ expression in immature, CD45^dim^ thymocytes as a marker for T-cell commitment that precedes acquisition of CD1a. Our results demonstrate that CD44^−^CD1a^−^ thymocytes have initiated TCR Dβ–Jβ and Vβ–Jβ rearrangements and are T-lineage restricted. These results explain why the CD34^+^CD1a^−^ thymocyte population from previous studies—considered to be uncommitted thymocytes—is in fact heterogeneous with mixed CD44^dim^ and CD44^−^ expression (8, 16). The multi-lineage potential of CD34^+^CD44^+^ human thymocytes has been suggested (27), but this study did not consider CD44^dim^ expression. Our study places CD44^dim^ expression in the context of other T-cell development markers in immature CD45^dim^ thymocytes and provides first functional evidence that loss of CD44^dim^ marks human T-cell commitment *in vivo*.

**Figure 7:**
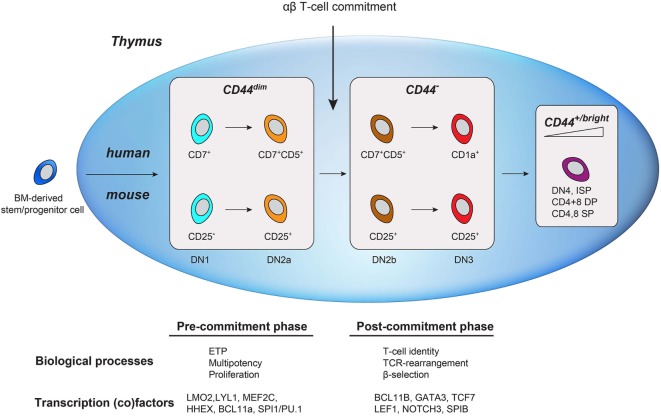
**Schematic comparison of human and mouse early T-cell development in the thymus**. Bone marrow-derived lymphoid progenitors enter the thymus (depicted as a blue oval) and exhibit dim CD44 expression. T-cell commitment is marked by the downregulation of CD44. As cells mature, they regain CD44 expression.

Similar to human early T-cell development, the major transcriptional change in murine T-cell development—well before the DP stage—segregates uncommitted from αβ T-cell committed thymocytes (2, 23). In murine thymocytes, this well-characterized transition occurs in the second CD4/8 DN stage (DN2; CD44^+^CD25^+^), before the TCRβ checkpoint in DN3 (CD44^−^CD25^+^) (14, 32). T-cell commitment is defined by the transition from DN2a to DN2b that is marked by the initial reduction of CD44 surface expression (2, 23). This reduction of CD44 expression in mice is conserved in humans (from CD44^dim^ to CD44^−^) and marks T-cell commitment. This suggests conservation of a functional role for CD44 in uncommitted thymocytes. CD44 is an glycosylated adhesion and migration molecule with several isoforms due to alternatively spliced exons in the extracellular domain (33). The standard CD44 isoform (CD44s) is expressed on hematopoietic (stem) cells (as well as many other cell types) and is required for HSC niche formation and quiescence during early hematopoietic development. In addition to its expression on hematopoietic stem/progenitor and ETP cells, CD44 is highly re-expressed on mature lymphocytes (Figure 7) and functions in the homing to secondary lymphoid organs. In the thymus, laminin-5-induced CD44 cleavage by metalloproteinase-14 also directs the migration of mature thymocytes within the medulla as well as the exit from the medulla (34). The rare population of uncommitted CD44^dim^ ETPs is present in subcapsular clusters in the cortex of the thymus, suggesting that CD44 also directs thymocyte precursors from the bone marrow to the thymus, entry into the thymus, and potentially intrathymic migration during early T-cell development (35, 36).

In T-cell acute lymphoblastic leukemia (T-ALL) and other T-cell malignancies, the relevance of CD44 expression is unknown. In light of the findings here, the CD44^dim^ expression on normal uncommitted ETPs may represent the stage of the immature subtype of T-ALL also referred to as ETP-ALL, a subtype characterized by expression of many stem cell genes. In many other cancer types and leukemias, CD44 has gained clinical interest because CD44s and variant isoforms (CD44v) feature “stemness” potential. Currently, multiple anti-CD44-based therapeutic approaches are investigated for their ability to target leukemia- or cancer-initiating cells (LIC/CIC) with this “stemness” potential (33).

By adding CD44, we have expanded the immunophenotypes of the different phases of early T-cell development in the human thymus (Figure 7). CD44^dim^CD45^dim^ human thymocytes reflect uncommitted ETP cells that are mainly characterized by the expression of transcription factors from alternative blood lineages, multi-lineage potential, and the absence of *TCRB* rearrangements. Loss of CD44^dim^ marks T-cell commitment that coincides with the upregulation of T-cell specific transcription factors (e.g., *GATA3, BCL11B, TCF7*), loss of alternative cell fate potential, and the initiation of *TCRB* rearrangements. Next, the thymocytes acquire CD1a and complete their *TCRB* rearrangements. In conclusion, combining CD44 and CD1a surface markers in CD45^dim^ thymocytes (in addition to other markers) is important in the characterization of pre- and post-committed human thymocytes and will help to better understand human T-cell development and T-cell commitment.

## Ethics Statement

Human UCB was obtained from consenting mothers after delivery at local hospitals. Thymi were obtained as surgical tissue discards from infants 2–9 months of age undergoing cardiac surgery at Erasmus MC Rotterdam. This study was carried out in accordance with the recommendations of the Institutional Review Board of the Erasmus MC Rotterdam with written informed consent from the parents or legal guardians of all subjects. All subjects gave written informed consent in accordance with the Declaration of Helsinki. The protocol was approved by the Institutional Review Board of the Erasmus MC Rotterdam.

## Author Contributions

KC-B, RM, and JM designed the study and wrote the manuscript; KC-B and RM performed the experiments; YL analyzed the bioinformatics data; EV flow sorted the different cell populations; KP-O and FS collected and provided the human thymocytes; and TW and AL performed V-D-J rearrangement experiments. JM and RP supervised the study. All the authors read and approved the paper and declared no competing financial interests.

## Conflict of Interest Statement

The authors declare that the research was conducted in the absence of any commercial or financial relationships that could be construed as a potential conflict of interest.

## Footnotes

^1^https://www.ncbi.nlm.nih.gov/geo/query/acc.cgi?acc=GSE79379.

^2^http://www.broadinstitute.org/gsea.
