# Supplementary material for: Loss of CD44dim Expression from Early Progenitor Cells Marks T-Cell Lineage Commitment in the Human Thymus
Source: Front Immunol. 2017 Jan 20;8:32. doi: 10.3389/fimmu.2017.00032 (PMC5247458; doi:10.3389/fimmu.2017.00032)
Supplement: Supplementary file 1 [file Data_Sheet_1.PDF]

Loss of CD44^{dim} expression from early progenitor cells marks T-cell lineage commitment in the human thymus

Kirsten Canté-Barrett[1,2,5], Rui D. Mendes[2,5], Yunlei Li[2], Eric Vroegindeweij[1,2], Karin Pike-Overzet[3], Tamara Wabeke[4], Anton W. Langerak[4], Rob Pieters[1,2], Frank J.T. Staal[3], and Jules P.P. Meijerink*[1,2]

[1]Princess Máxima Center for Pediatric Oncology, Utrecht, The Netherlands;
[2]Department of Pediatric Oncology/Hematology, Erasmus Medical Center-Sophia Children's Hospital, Rotterdam, The Netherlands;
[3]Department of Immunohematology and Blood Transfusion, Leiden University Medical Center, Leiden, The Netherlands;
[4]Department of Immunology, Erasmus Medical Center, Rotterdam, The Netherlands.
[5]Co-first author

*Correspondence: Jules P.P. Meijerink, PhD
Princess Máxima Center for Pediatric Oncology
Uppsalalaan 8
3584 CT Utrecht
The Netherlands
e-mail: j.meijerink@prinsesmaximacentrum.nl

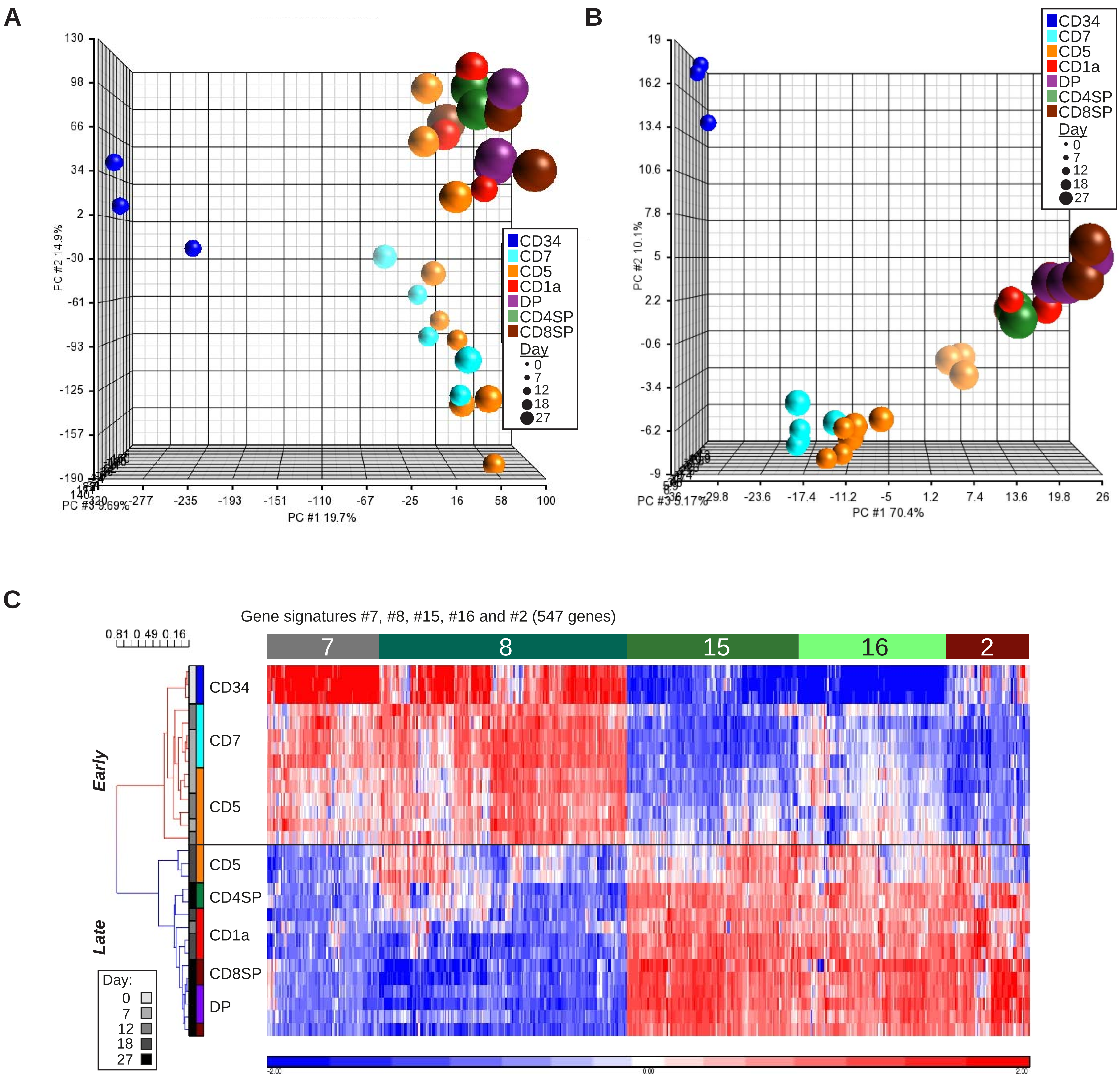

Supplemental Figure S1. Related to Figure 1. Consecutive stages of *in vitro* T-cell differentiation can be divided into clusters with distinct gene expression. Principal component analysis of gene expression data of 29 sorted populations using all probesets (n=54715) of the array (**A**) or using 547 genes (gene signatures #7,# 8, #15, #16, #2) (**B**). (**C**) Hierarchical clustering analysis (Pearson average method) of the different sorted populations based on the 547 genes that discriminate early (characterized by gene signatures #7 and #8) from late (characterized by gene signatures #15, #16 and #2) T-cell programs. The clustering obtained is similar to Fig. 1C where 1387 genes were used.

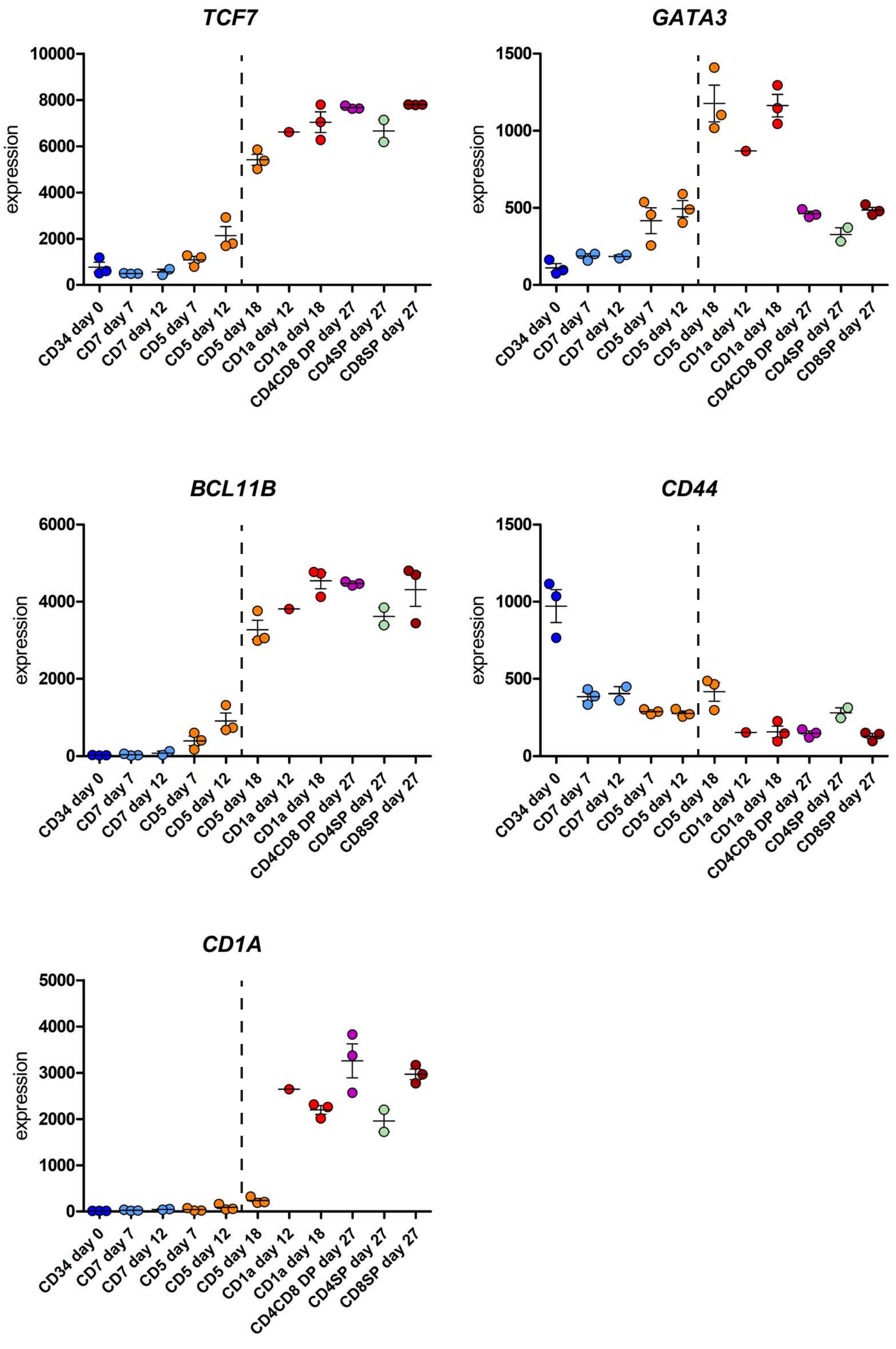

Supplemental Figure S2. Related to Figure 1. Examples of gene expression in consecutive stages of *in vitro* T-cell differentiation. Gene expression of genes encoding T-cell specific transcription factors *TCF7*, *GATA3*, *BCL11B*, and the surface marker *CD1A* (from gene signature #15, Fig. 1D), as well as *CD44* gene expression (from gene signature #8, Fig. 1D). Each data point represents a cell population sorted on a certain day and stage of *in vitro* T-cell differentiation (see table in Fig. 1A). The vertical dashed line divides early (left) and late (right) T-cell differentiation populations, representing pre- and post- T-cell commitment.

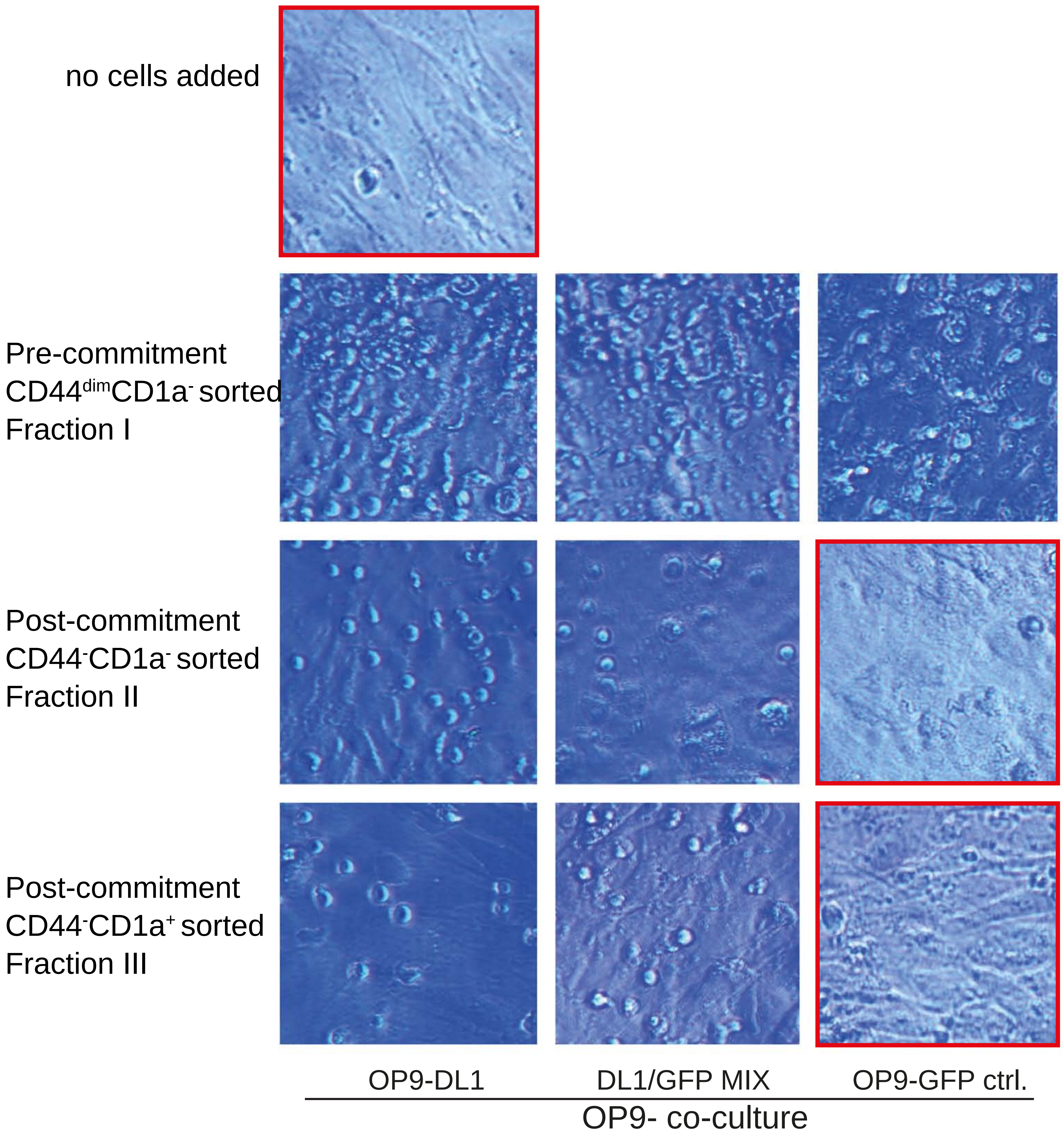

Supplemental Figure S3. Related to Figure 6. Bright field microscopic images of sorted and co-cultured human thymocyte populations I-III. Images (200x) were generated after 7 days of co-culture. OP9-DL1 stromal cells support T- and other lineage differentiation, whereas OP9-GFP control cells do not support T-lineage differentiation (the red outlines indicate images with only OP9 stromal cells). The middle column represents co-cultures on OP9-DL1:GFP 1:1 mixed cells.

Supplemental Tables

Table S1: Gene Symbols in each of the 16 Gene Signatures from Figure 1C-E

Gene Signature	Gene Symbol
1	JHDM1D
1	BTG2
1	NBEA
1	PFKFB2
1	HIST1H2AC
1	ARID5B
1	PELI2
1	ERN1
1	BTBD11
1	ZNF704
1	RORA
1	MAFB
1	LOC283070
1	CAMK1D///LOC283070
1	CAMK1D
1	SPOCK2
1	DDIT4
1	TP53INP1
1	HIST2H2BE
1	CREBRF
1	AMY1A///AMY1B///AMY1C///AMY2A
1	LOC728392
1	DCHS1
1	PCMTD1
1	BRWD1
2	TRERF1
2	DNTT
2	ITGB2-AS1
2	LINC00340
2	GAL3ST4
2	OTTHUMG00000166495///RP11-728F11.1
2	MBD5
2	LBH
2	SCN3A
2	IL10RA
2	FAIM3
2	CRIP1
2	OTTHUMG00000178878///RP11-214C8.5
2	PCDH9
2	NEGR1
2	ETS1
2	BACH2
2	PGM2L1
2	DUSP16
2	TANC1
2	ITGA6
2	ARRDC4
2	AGBL3
2	PRSS2///PRSS3
2	SCN7A
2	PDE4D
2	TUBB2A
2	ARHGAP29
2	SIPA1L2
2	ID2///ID2B
2	CDKN2D
2	S1PR1
2	IKZF3
2	RBMS3
2	NSG1
2	NLGN4X
2	LRRN3
2	CD8A
2	NINL
2	CD8B///LOC100996919
2	EFNB2
9	S100A8
9	S100A9
9	DEFA1///DEFA1B///DEFA3
9	TACSTD2
9	CHI3L1
9	CD24
9	CSGALNACT1
9	MS4A3
9	CSTA
9	ACTN1
9	PRG2
9	CLC
9	RNASE3
9	S100P
9	RNASE2
9	CTSG
9	ELANE
9	CEACAM6
9	CST7
9	PRTN3
9	AZU1
9	CEACAM8
9	BPI
9	DEFA4
9	SLPI
9	ANXA3
9	ETV5
9	TNFRSF4
9	TNFRSF18
9	PRF1
9	VPREB1
9	CTSW
9	SVOPL
9	GPA33
9	PPIF
9	ELOVL6
9	CTHRC1
9	CSRP2
9	TNFSF4
9	PRR5L
9	GCNT1
9	RAB27B
9	GZMA
9	NKG7
9	DNMT3B
9	TESC
9	ARRB1
9	APOBEC3B
9	IGLL1
9	GUSBP11///IGLL1///IGLL3P
9	COL5A2
9	SLC35F3
9	CISH
9	CFH///CFHR1
9	TFEC
9	SLC39A14
9	NLN
9	FKBP4
9	TTLL12
9	PUS7
9	OSM
9	H19///MIR675
9	LIN28B
10	AGR2
10	MS4A1
10	KLRD1

2	VCAN
2	LOC100287598
2	ID1
2	RASSF6
2	SEC31B
2	COX6A1
2	GBP1
2	KANSL1-AS1
2	GAS2
2	PALLD
2	HNRNPLL
2	ANKRD36BP2///LOC101060554
2	PLEKHG1
2	NCALD
2	SYNM
2	GRK5
2	RDH10
2	ALOX5
2	FHIT
2	TRERF1
2	DNTT
2	ITGB2-AS1
2	LINC00340
2	GAL3ST4
2	OTTHUMG00000166495///RP11-728F11.1
2	MBD5
2	LBH
2	SCN3A
2	IL10RA
2	FAIM3
2	CRIP1
2	OTTHUMG00000178878///RP11-214C8.5
2	PCDH9
2	NEGR1
2	ETS1
2	BACH2
2	PGM2L1
2	DUSP16
2	TANC1
2	ITGA6
2	ARRDC4
2	AGBL3
2	PRSS2///PRSS3
2	SCN7A
2	PDE4D
2	TUBB2A
2	ARHGAP29
2	SIPA1L2
2	ID2///ID2B
2	CDKN2D
2	S1PR1
2	IKZF3
2	RBMS3
2	NSG1
2	NLGN4X
2	LRRN3
2	CD8A
2	NINL
2	CD8B///LOC100996919
2	EFNB2
2	VCAN
2	LOC100287598
2	ID1
2	RASSF6
2	SEC31B
2	COX6A1
2	GBP1
2	KANSL1-AS1
2	GAS2
2	PALLD
2	HNRNPLL
2	ANKRD36BP2///LOC101060554
10	IL18RAP
10	GNLY
10	XCL1
10	ITGAM
10	SAMD3
10	KLRB1
10	KLRC4-KLRK1///KLRK1
10	CRTAM
10	PTGDR
10	PDE7B
10	EPHA4
10	KLRC3
10	MAF
10	KLRC1///KLRC2
10	IL2RB
11	FCRL4
11	SIGLEC6
11	UGCG
11	CTA-250D10.23///OTTHUMG00000172730
11	C1orf186///LOC100505650
11	TBC1D9
11	CDH1
11	FGL2
11	ENTPD1
11	MPEG1
11	FGD2
11	LGMN
11	CACNA2D3
11	SLAMF7
11	LY9
11	PTPRJ
11	MCOLN2
11	CCR2
11	TGFBI
11	NAPSB
11	MYCL1
11	TLR10
11	C5orf20///TIFAB
11	TIFAB
11	LYZ
11	IL6R
11	MRC1
11	MNDA
11	P2RY14
11	TYROBP
11	FCER1G
11	CCR1
11	CD36
11	CTSZ
11	MS4A7
11	CD86
11	NCF2
11	GSN
11	AMICA1
11	SLC41A2
11	DNASE1L3
11	TICAM2///TMED7-TICAM2
11	GAS2L3
11	RGS18
11	IDH3A
11	CD48
11	IGJ
11	TRIB2
11	SMIM14
11	RNASE6
11	CLCN5
11	GPR18
11	LOC158402
11	FGR
11	EPHB1
11	OTTHUMG00000175583///RP11-4O1.2
11	ARL4C

2	PLEKHG1
2	NCALD
2	SYNM
2	GRK5
2	RDH10
2	ALOX5
2	FHIT
3	DENND1B
3	PAPD4
3	U2SURP
3	SF1
3	SREK1
3	LOC100996467
3	MALAT1
3	CLK4
3	OTTHUMG00000168768///RP11-463O12.5
3	KMT2A
3	CAPZA1
3	ZMYM2
3	CEP95
3	B2M
3	IFNGR1
3	EIF5///SNORA28
3	MORF4L2
3	NFAT5
3	C10orf118
3	PCNX
3	AC092620.2///OTTHUMG00000153633
3	TGFBR1
3	INPP5D
3	MIR612///NEAT1
3	WDR11
3	OTTHUMG00000168867///RP11-305O6.3
3	LRRFIP1
3	CELF2
3	LONRF1
3	ANKRD28
3	ZNF274
3	LOC100506312
3	SEC61B
3	NKTR
3	PIK3C2A
3	RAB18
3	GNAS
3	PELI1
3	UBE2B
3	TBRG1
3	LINC00597
3	SAMD4B
3	DAPK1-IT1
3	PGAP1
3	MLLT3
3	SPATA13
3	RGCC
3	BCL6
3	KLF4
3	RGS1
3	LOC100507577///LONP2
3	ZBTB20
3	OTTHUMG00000175755///RP11-553L6.5
3	LOC100131564
3	PPP2R5C
3	HECA
3	LOC100996653
3	APBB1IP
3	TNFAIP3
3	STK4
3	PRO2852
3	ZFAND6
3	ZNF638///ZNF638-IT1
3	RBM5
3	RUNX1-IT1
11	GPR183
11	CLECL1
11	FAM105A
11	SAMHD1
11	CLEC4A
11	ITGB7
11	BLNK
11	STX7
11	IFI44L
11	OAS1
11	CRYBG3
11	ABHD6
11	KYNU
11	CCR6
11	HCK
11	GAS6
11	IGK///IGKC
11	DAB2
11	MS4A4A
11	HDAC9
11	CLEC12A
11	KMO
11	IRF8
11	CYBB
11	DTX4
11	ADRB2
11	THBD
11	RBM47
11	ATP10D
11	CD180
11	GPR114
11	GAPT
11	PAX5
11	ARHGAP18
11	KIAA0930
11	SUCNR1
11	LY86
12	GZMB
12	NME8
12	TCL1A
12	CMKLR1
12	PTPRS
12	IL18R1
12	SOGA2
12	TPM2
12	CYAT1///IGLC1///IGLV1-44
12	IGLC1
12	LAMP5
12	LOC285972
12	SEL1L3
12	IL3RA
12	GNG7
12	LHFPL2
12	ZDHHC14
12	BANK1
12	CCDC50
12	FZD3
12	FCRLA
12	IFNLR1
12	SPIB
12	IRF7
12	FAM129C
12	SULF2
12	LILRA4
12	CLEC4C
12	PAPLN
13	CDH17
13	IL1R2
13	IGSF6
13	CLEC10A
13	LGALS2
13	HLA-DQB1

3	CCAR1
3	GGNBP2
3	PDCD6
3	RPS27
3	RPS16P5
3	TLE4
3	LOC100996866
3	IKZF1
3	GUSBP3///GUSBP9///LOC100653061///LOC101060519
3	MSI2
3	RHOH
3	LOC100190986///LOC101060564
3	LINC00342
3	FLJ31306
3	FLJ38379
3	TCF12
3	RAB11FIP3
3	ZNF124
4	C1orf54
4	GGTA1P
4	FCER1A
4	LGALS3
4	HLA-DRB1///HLA-DRB3///HLA-DRB4///HLA-DRB5///LOC100507709///LOC100507714
4	SERPINB2
4	THEMIS2
4	SETBP1
4	SERPINB9
4	EMP1
4	HLA-DPA1
4	CD74
4	CSF2RB
4	HLA-DOA
4	AHNAK
4	HLA-DRB1///HLA-DRB3///HLA-DRB4///HLA-DRB5///LOC100507709///LOC100507714///LOC101060779
4	DUSP1
4	RGS2
4	KLF6
4	SIK1
4	ARRDC3
4	ZBTB24
4	PLK2
4	KLF2
4	PPP1R15A
4	DUSP4
4	ETNK1
4	EGR3
4	PTGS2
4	NR4A2
4	FOSB
4	FOS
4	VIM
4	SGK1
4	RASGEF1B
4	LOC284454
4	ANXA1
4	ITPKB
4	NASP
4	EGR2
4	LAIR2
5	VWF
5	EFEMP1
5	MGP
5	MAFIP
5	ELL2
5	FN1
5	HBEGF
5	ABLIM1
5	NRIP1
5	PIK3R1
5	MYH10
5	OTTHUMG00000182869///RP11-420K14.2
5	FLJ13197

13	HLA-DQA1
13	AGPAT9
13	CLIC2
13	CLEC7A
13	IL13RA1
13	CST3
13	AXL
13	OTTHUMG00000173465///RP11-389C8.2
13	MS4A6A
13	GEM
13	PKIB
13	S100B
13	TLR7
13	SERPINF1
13	CCR5
13	JAZF1
13	MARCH1
13	CPVL
13	IDO1
13	FCGR2B
14	TASP1
14	GXYLT2
14	TRDC
14	YME1L1
14	CD3D
14	TRAT1
14	GPR171
14	GPR65
14	LPXN
14	CDK2AP2
14	VANGL1
14	VASH2
14	CD7
14	HES4
14	ICOS
14	MYO1B
14	CEP41
14	SLC35B3
14	TMEM251
14	ATG4C
14	VPS41
14	ATP6V1D
14	BPNT1
14	KIAA1841
14	PPHLN1
14	C17orf62
14	PWWP2A
14	CBR4
14	WDR67
14	DCAF12
14	LRIF1
14	ST8SIA4
14	DNA2
14	LOC389831
14	HES1
14	GIMAP7
14	IL17RA
14	DPP4
14	IFNAR1
14	PAQR8
14	TRIM13
14	ACSL3
14	SHCBP1
14	UMODL1
14	PHF20L1
14	STK38L
14	CEP19
14	C9orf64
14	HYLS1
14	ATP6V1C1
14	ERCC4
14	ARL13B

5	ZBTB16	14	KBTBD7
5	CDKN1C	14	HDHD1
5	PXDN	14	FBXO5
5	HIST3H2A	14	NCAPG2
5	OCLN	14	SKA3
5	HLA-DRB4	14	CCNF
5	XIST	14	TROAP
5	FOXO3///FOXO3B	14	AURKB
5	MXI1	14	CASC5
5	DGKE	14	NUSAP1
5	PPP1R3B	14	TMPO
5	ABCG1	14	CCDC15
5	RBM38	14	SGOL2
5	SHISA2	14	GTSE1
5	GNL1	14	CDCA2
5	BAG3	14	E2F7
5	SPTBN1	14	E2F8
5	CREM	14	KIF18B
5	KBTBD2	14	MKI67
5	IL6ST	14	KIF23
5	SKIL	14	ECT2
5	RIT1	14	CENPF
5	HIST2H2AA3///HIST2H2AA4	14	NUF2
5	PIK3IP1	14	FANCI
5	ANKDD1A	14	KIF15
5	CCNL1	14	KIF18A
5	JUN	14	ESPL1
5	MEX3C	14	KIFC1
5	CSRNP1	14	BUB1B
5	BCL10	14	STIL
5	EPC1	14	KIF14
5	BTG1	14	CENPE
5	KRAS	14	DEPDC1
5	CHMP3///RNF103///RNF103-CHMP3	14	KIF2C
5	KLHL24	14	HJURP
5	MIR4800///MXD4	14	CDCA8
5	OTUD1	14	CCNB2
5	CA2	14	CENPA
5	RIN2	14	NEK2
5	LILRB2	14	KIF11
5	CPQ	14	KIF4A
5	ADAM8	14	BUB1
5	SQSTM1	14	RRM2
5	IL8	14	TPX2
5	TRIB1	14	RACGAP1
5	CCL3///CCL3L1///CCL3L3///LOC101060267	14	MELK
5	ZFP36	14	SPAG5
5	TSC22D3	14	NCAPG
5	METRNL	14	SRSF1
5	ATF3	14	NDC80
5	NAMPT	14	SPDL1
5	RC3H2	14	CENPN
5	MAFF	14	C9orf40
5	POLB	14	CKAP2L
5	CUL1	14	ARHGAP11A
5	HEMGN	14	FOXM1
5	PER1	14	NCAPH
5	PCIF1	14	RAD54L
5	CSNK1E///LOC100996460	14	BIRC5
5	CUL4A	14	CCDC167
5	STAT3	14	CCNE2
5	FAM3C	14	ERCC6L
5	RAPGEF2	14	PARPBP
5	RNF130	14	C11orf82
5	MECP2	14	KNSTRN
5	SRSF11	14	CDCA5
5	NLRP1	14	ASF1B
5	MED23	14	AURKA
5	ST3GAL1	14	CDKN3
5	ZNF638	14	MND1
5	IREB2	14	NIF3L1
5	MIR22///MIR22HG	14	MTFR2

5	PPP1R16B
5	FLJ43663
5	ISG20
5	PIM2
5	PDE4B
5	SOCS3
5	LPGAT1
5	HLA-E
5	SMAD3
5	RNF144B
5	IDS
5	ERO1LB
5	ACVR2A
5	TMEM200A
5	IRAK2
5	GABARAPL1
5	SLC2A3
5	SYTL3
6	HAS1
6	CTSL1
6	NINJ1
6	CXCL3
6	TM4SF1
6	INHBA
6	CXCL1
6	AQP9
6	CCL2
6	ICAM1
6	PHLDA1
6	TNFRSF9
6	IL6
6	ZNF709
6	C10orf10
6	RHOB
6	IER3
6	C15orf48
6	BCL2A1
6	TLN1
6	HIST1H2BC
6	TPP2
6	IL1RL1
6	CNST
6	USP36
6	MAX
6	FTH1
6	USP15
6	GFOD1
6	DOCK7
6	AREG///AREGB
6	C7orf73///SLC13A4
6	SLC8A3
6	TPM3
6	SOD2
6	GABPB1
6	STX11
6	CXCL2
6	FAM198B
6	CYLD
6	RELB
6	BCL3
6	NBL1
6	SLCO3A1
6	SMURF1
6	SEMA6A
6	SDK2
6	MINOS1-NBL1///NBL1
6	SERAC1
6	EIF4G3
6	NFKB2
6	USP53
6	ERMN
6	TCF25
14	TCF19
14	HIRIP3
14	SPC24
14	RTP4
14	RBL1
14	TOP2A
14	FAM72A///FAM72B///FAM72C///LOC101060656
14	UHRF1
14	WDR76
14	INTS7
14	CDK1
14	DLGAP5
14	TTK
14	UBE2C
14	CDCA3
14	KIF20A
14	CEP55
14	HMMR
14	ANLN
14	BRIP1
14	ERI2
14	DIAPH3
14	C1orf112
14	SMC2
14	DEPDC1B
14	UBE2T
14	CCNA2
14	CDC20
14	CCNB1
14	OIP5
14	SPC25
14	PKP4
14	BRCC3
14	CDC25A
14	FBXO22
14	SLC25A19
14	POLR1B
14	IDH1
14	HK2
14	GIMAP6
14	GIMAP8
14	GIMAP1
14	NABP1
14	NRARP
14	GAS1
14	DCPS
14	ARL11
14	SGK223
14	STAMBPL1
14	OPN3
14	NLRP2
14	PSPH
14	FAM173B
14	CHEK2
14	ICMT
14	POLR3K
14	C2orf43
14	RAD51C
14	GART
14	CSE1L
14	OTTHUMG00000159786///RP11-85F14.5
14	TRMT1L
14	METTL13
14	UBE2L3
14	EPDR1
14	RPE
14	BRI3BP
14	RMI1
14	KIAA1524
14	GINS1
14	ARFIP1
14	SHQ1

6	GNG4
6	KSR1
6	SKI
6	MEG3
6	AVP
6	THBS1
6	HBA1///HBA2
6	NR4A3
6	BMP6
6	CDC42
6	LOC100507672
6	BEX5
6	CDC14B
6	NEDD4L
6	HBG1///HBG2
6	DLK1
6	PPBP
6	G0S2
6	TMEM163
6	TNIK
6	THSD7A
6	LIMS3///LIMS3L
6	ARHGEF12
6	FOXO1
6	FAM65C
6	PDZD2
6	EREG
6	HBB
6	LOC254057
6	PEAR1
6	FCHO2
6	CRHBP
6	MYCT1
6	DNAJC6
6	BEX2
6	ACSL1
6	BEX1
6	H1F0
6	KLF3
6	LRP5L
6	PRKACB
6	NLK
6	C7orf41
6	MAGI2-AS3
6	OTTHUMG00000176268///RP11-18F14.2
6	SLC35D2
6	LOC285812
6	PTX3
6	GBP2
6	ABCB1
6	CEBPB
6	FGD5
6	ABHD17C
6	MPP7
6	IRS2
6	PPM1H
6	PLOD2
6	RBPMS
6	HLF
6	PBX1
6	CALN1
6	GPR126
6	GNAI1
6	ALDH1A1
6	TNFRSF10A
6	GATA2
6	TNFRSF10D
6	CNRIP1
6	ITGA9
6	TAL1
6	TCF7L2
6	DACH1

14	TARP///TRGC2
14	RAD51
14	DHFR
14	BRCA1
14	MCM8
14	TK1
14	GUCY1B3
14	TAF5
14	TDP1
14	TUBG1
14	PAGR1
14	XRCC4
14	SRSF10
14	CENPM
14	WDHD1
14	PANK1
14	LCMT2
14	PSAT1
14	EXO1
14	C12orf4
14	C18orf54
14	MED11
14	EXOSC3
14	GEMIN6
14	UMPS
14	TRIP13
14	CDC6
14	MCM10
14	CHAC2
14	CLUAP1
14	RAD54B
14	SAPCD2
14	GINS4
15	CD1C
15	SMPD3
15	CD1E
15	HHIP-AS1
15	CHI3L2
15	SYNE2
15	TNRC6C
15	ARPP21
15	LOC100130503
15	DOCK9
15	OTTHUMG00000176181///RP11-119F7.5
15	CCR9
15	GSDMB
15	CTC1
15	HS3ST3B1
15	ZBTB1
15	FAM69A
15	RUFY3
15	CCNG2
15	PVRIG
15	NOTCH3
15	HDAC4
15	LRRC28
15	ACPL2
15	LINC00226
15	CR2
15	ZC3H6
15	NKD2
15	GLRB
15	OTTHUMG00000017520///RP11-144L1.4
15	CX3CR1
15	LOC101060391
15	CCR8
15	TMSB15A///TMSB15B
15	SLC16A10
15	CD1B
15	RAG2
15	AQP3
15	CD1A

6	LRP6
6	ZNF521
6	NPR3
7	SOCS2
7	ARMCX1
7	MEST
7	CXCR7
7	HSPA1A///HSPA1B
7	C11orf96
7	GSTM3
7	FHL1
7	SNTB1
7	BSPRY
7	EPCAM
7	HBD
7	UCHL1
7	NRIP3
7	PDE4DIP
7	MATR3///SNHG4
7	AC005682.5///OTTHUMG00000152563
7	TMEM220
7	GYPC
7	FAM171B
7	ADM
7	SMIM3
7	CRYGD
7	RIPK2
7	ZBTB8A
7	GBP4
7	DRAM1
7	MPL
7	ICA1
7	CMBL
7	SYNGR1
7	FAM69B
7	TCTEX1D1
7	SDPR
7	OTTHUMG00000020937///RP11-251M1.1
7	CPA3
7	KBTBD11
7	SLC22A15
7	RAB13
7	GRB10
7	SLC2A5
7	ELK3
7	PVRL2
7	SORL1
7	PROK2
7	GPR27
7	SNORD3A///SNORD3B-1///SNORD3B-2///SNORD3C///SNORD3D
7	PRSS2
7	MSRB3
7	NAP1L3
7	TPM1
7	HMGA2
7	NUDT11
7	CABLES1
7	CKAP4
7	RAMP1
7	TFPI
7	IL1RAP
7	IRAK3
7	GBP5
7	FOSL2
7	SLC22A4
7	TRH
7	TTC7B
7	HOXB2
7	ME3
7	CHRM3
7	MECOM
7	MEIS1
15	GRAP2
15	CD3E
15	SLC7A3
15	RAPGEF5
15	ATP6AP1L///FLJ41309
15	SRPK2
15	TRBC1
15	TRBC2
15	MAL
15	ID3
15	UBASH3A
15	TLR5
15	TSHR
15	IRS1
15	HHIP
15	PTCRA
15	SLC8A1-AS1
15	PAFAH2
15	RAG1
15	CD1D
15	MME
15	PLCL1
15	SH3TC1
15	SPRED2
15	BCL2L11
15	FOXO6///FOXO6
15	CYP2U1
15	PPP1R1C
15	LOC79015
15	STAG3
15	CRNDE
15	ADAMTS1
15	WT1
15	LOC283788
15	LOC728613
15	SLC16A6
15	IPP
15	TFDP2
15	SBK1
15	POU2AF1
15	ITK
15	RASGRP1
15	CAMK4
15	TCF7
15	PCDH10
15	PTPRK
15	CD96
15	GATA3
15	IL32
15	TMOD2
15	PLCG1
15	NMT2
15	P2RX5
15	IKZF2
15	FAM63B
15	DGKA
15	RGPD1///RGPD2
15	LAT
15	BCL11B
15	ZAP70
15	THEMIS
15	CD247
15	SLFN5
15	LEF1
15	CD3G
15	LDLRAD4
15	AEBP1
15	SLC4A4
15	GALNT2
15	KIF3A
15	PARP8
15	RIMS3

7	PHTF1
7	LOC100505573
7	LATS2
7	MBOAT7
7	LPCAT2
7	AAED1
7	WHAMMP2///WHAMMP3
7	SEMA4C
7	KIT
7	HOPX
7	HOXA3
7	HOXA5
8	C11orf21
8	GSAP
8	GATM
8	STAP1
8	LYN
8	ALCAM
8	VCL
8	MEF2C
8	BTK
8	MGLL
8	B3GNT7
8	FAM49A
8	PLXNB2
8	PHACTR1
8	PLEK
8	BASP1
8	IRF5
8	DUSP6
8	CD300LF
8	RAB31
8	PIK3AP1
8	MYO1F
8	CTBP2
8	CDC42BPA
8	IPO11///LRRC70
8	DUSP10
8	C1orf186
8	RPS11
8	BHLHE40
8	SPRY1
8	NFIL3
8	KLF11
8	SPRY2
8	CD69
8	EGR1
8	DUSP5
8	FXYD5
8	CD44
8	OTTHUMG00000176545///RP11-510J16.3
8	MAML3
8	LAPTM5
8	CRIM1
8	IGHM
8	SNX9
8	FLT3
8	HLX
8	TCF4
8	NFKBIZ
8	CAPN2
8	MAP3K8
8	ADAM28
8	MYLIP
8	KLF9
8	LILRA2
8	HPGDS
8	CD302///LY75-CD302
8	HHEX
8	TNS3
8	SH3RF1
8	ANKRD33B
15	FYB
15	YPEL1
15	KIAA0226L
15	NEDD9
15	CD72
15	ATM
15	IPCEF1
15	APBA2
15	SERHL2
15	CD79A
15	EBF1
15	CDH2
16	KBTBD6
16	FRMD4A
16	DLEU2
16	NAPEPLD
16	EVL
16	LOC100506776
16	SLC4A7
16	NREP
16	KATNBL1
16	AHRR
16	FANCA
16	POLQ
16	ZNF519
16	LOC100507312
16	ASPM
16	ESCO2
16	FAM111B
16	ATAD2
16	RHOU
16	LYST
16	LOC284757
16	GTF2H2B
16	TXLNG2P
16	DSERG1
16	PLGLB1///PLGLB2
16	SEPT1
16	HOMER1
16	RRN3P3
16	UGP2
16	LOC100996511
16	MIR181A2HG
16	GOLGA2P5
16	OTTHUMG00000183913///RP11-932O9.10
16	EPM2AIP1
16	C1orf132
16	CD28
16	DHFRL1
16	SUV420H1
16	AKAP2///PALM2-AKAP2
16	TRAF3IP3
16	SATB1
16	INPP4A
16	ITGAL
16	CDC25B
16	SH3KBP1
16	NDST3
16	HNRNPR
16	ARHGAP19
16	GRSF1
16	CEP70
16	WDR7
16	CEP78
16	STAU2
16	TBCD
16	LRRC1
16	WBP1L
16	SELPLG
16	JPH1
16	PRKCA
16	BIK

8	NLRP3
8	GNG11
8	ARSD
8	FNDC3B
8	SLC25A13
8	GOLIM4
8	TIMP1
8	NEK3
8	C10orf54
8	DUSP3
8	TNFRSF1B
8	IL18
8	ITPRIPL2
8	CEBPD
8	FAM46A
8	CTNNA1
8	LMO2
8	TNFRSF10B
8	CFD
8	SLC7A5
8	BATF
8	LOC729680
8	EIF4EBP1
8	TRIM6
8	MGST1
8	MPO
8	SLC22A16
8	FAH
8	BCAT1
8	MTHFD1L
8	TNFAIP2
8	TNFSF13B
8	CD33
8	PLA2G4A
8	MOB3B
8	KCNK17
8	GCSAML
8	NFE2
8	HSH2D
8	LAT2
8	P2RY2
8	SIGLEC17P
8	ACY3
8	S100Z
8	PAM
8	CD34
8	C11orf74
8	PYGL
8	CLDN10
8	CYB561
8	CLEC11A
8	MYCN
8	SPON1
8	ANGPT1
8	PTPRD
8	SPINK2
8	KIAA0125
8	EFHC2
8	MCTP2
8	AP001171.1///OTTHUMG00000074606
8	ERG
8	IGFBP7
8	ARHGEF40
8	MYLK
8	LYL1
8	PRAM1
8	SERPINB1
8	ATP8B4
8	XBP1
8	LAMC1
8	C19orf77
8	MAP7
16	OTTHUMG00000178927///RP11-196G18.23
16	CHST2
16	STS
16	RHBDD1
16	OXNAD1
16	MNS1
16	PSRC1
16	CDC25C
16	LOC100288637
16	FBXO43
16	MYBL1
16	LEF1-AS1
16	NEIL3
16	LOC100506100
16	KIAA0922
16	MTA3
16	CEP128
16	ITPR2
16	TCFL5
16	STK11IP
16	ZNF280D
16	APOLD1
16	ZEB1-AS1
16	CD2
16	LOC100507600
16	CAPSL
16	MGAT4A
16	NDFIP2
16	HIVEP3
16	PCSK5
16	IGHG1
16	IGH///IGHA1///IGHA2///IGHD///IGHG1///IGHG3///IGHG4///IGHM///IGHV3-23///IGHV4-31
16	MAP1A
16	RCAN1
16	SH2D1A
16	PBK
16	IL7R
16	ADA
16	LCK
16	LIG4
16	CDKN2C
16	E2F2
16	NETO2
16	GAS7
16	FXYD2
16	IGF2R

8	PROSER2
8	HTR1F
8	SPARC
8	SHANK3
8	F2RL1
8	ZC3H12C
8	CNKSR3
8	MN1
8	TUSC1
8	C1QTNF4
8	C9orf43
8	KIF13A
8	BAALC
8	PROM1
8	IQCJ-SCHIP1///SCHIP1
8	IL1B
8	B3GNT5
8	NIPAL2
8	DEPTOR
8	STOM
8	SERPINB6
8	CREG1
8	CD63
8	C17orf58
8	MYO5C
8	RAB3D
8	ASB9
8	SETD9
8	CYTL1
8	DPPA4
8	KCTD15
8	PDGFC
8	MATR3
8	CCND2
8	CPXM1
8	SLC39A8
8	SERPINE2
8	LAPTM4B
8	KIAA1211
8	STON2
8	LDLRAD3
8	KCNQ5
8	SLC27A2
8	KIF9
8	SLC35F2

Table S2: Gene set enrichment on gene expression of murine T-cell development, Figure 2A

Gene Signatures #7 and #8

Gene Symbol	Rank in gene list	Running ES	Core enrichment
RAB31	0	0.023	Yes
PLEK	2	0.043	Yes
HHEX	4	0.061	Yes
KIT	9	0.079	Yes
LYN	10	0.096	Yes
MEF2C	12	0.113	Yes
BTK	14	0.131	Yes
LYL1	15	0.147	Yes
ANGPT1	19	0.164	Yes
CD34	28	0.179	Yes
PLA2G4A	32	0.194	Yes
KIF13A	45	0.207	Yes
PLXNB2	54	0.220	Yes
ERG	63	0.233	Yes
MYO1F	69	0.245	Yes
PYGL	72	0.257	Yes
CTBP2	82	0.269	Yes
B3GNT5	99	0.280	Yes
CABLES1	101	0.291	Yes
IRF5	103	0.303	Yes
CD44	108	0.314	Yes
PIK3AP1	114	0.325	Yes
VCL	126	0.336	Yes
MYCN	133	0.346	Yes
MPO	142	0.357	Yes
ALCAM	148	0.367	Yes
NFE2	171	0.377	Yes
TCF4	284	0.381	Yes
SNX9	290	0.389	Yes
ZC3H12C	330	0.396	Yes
IL18	335	0.404	Yes
HOXA5	344	0.412	Yes
SLC25A13	354	0.420	Yes
FAM69B	355	0.428	Yes
CTNNA1	360	0.436	Yes
FAM49A	370	0.444	Yes
TRIM6	386	0.451	Yes
CNKSR3	392	0.459	Yes
LMO2	394	0.467	Yes
LAT2	452	0.472	Yes
HLX	467	0.479	Yes
P2RY2	501	0.485	Yes
IL1RAP	519	0.491	Yes
EIF4EBP1	523	0.498	Yes
ICA1	539	0.505	Yes
MEIS1	573	0.510	Yes
LPCAT2	595	0.516	Yes
ITPRIPL2	613	0.523	Yes
HPGDS	628	0.529	Yes
FLT3	631	0.536	Yes
CKAP4	636	0.542	Yes
PAM	649	0.549	Yes
GATM	669	0.555	Yes
ARMCX1	717	0.559	Yes
HMGA2	755	0.564	Yes
GSTM3	763	0.570	Yes
GOLIM4	779	0.576	Yes
CD33	892	0.577	Yes
NAP1L3	901	0.582	Yes
MAML3	963	0.585	Yes
MGST1	1076	0.586	Yes
GBP5	1077	0.592	Yes
SEMA4C	1117	0.595	Yes
DUSP3	1147	0.599	Yes
MSRB3	1181	0.603	Yes
IRAK3	1227	0.607	Yes
RAB3D	1254	0.611	Yes

Gene Signatures #2, #15 and #16

Gene Symbol	Rank in gene list	Running ES	Core enrichment
CD72	21	0.021	No
PARP8	369	0.016	No
TFDP2	399	0.026	No
ACPL2	587	0.027	No
SLC4A7	818	0.025	No
PAFAH2	851	0.032	No
STAU2	1238	0.022	No
KIF3A	1913	-0.002	No
ARRDC4	1914	0.003	No
CAPSL	2025	0.004	No
FAM69A	2152	0.004	No
SH3TC1	2312	0.002	No
FRMD4A	2418	0.002	No
SLC16A6	2503	0.003	No
SYNE2	2504	0.007	No
EBF1	3291	-0.023	No
STK11IP	3359	-0.023	No
ALOX5	3451	-0.023	No
RASSF6	3470	-0.021	No
NMT2	3613	-0.024	No
IL10RA	3730	-0.026	No
CEP70	3799	-0.025	No
PGM2L1	3814	-0.023	No
UGP2	4240	-0.039	No
GRK5	4308	-0.039	No
ATM	4523	-0.046	No
RUFY3	4632	-0.049	No
DUSP16	4635	-0.047	No
CD79A	4710	-0.048	No
GRSF1	4822	-0.051	No
DOCK9	4974	-0.055	No
HNRNPR	4991	-0.054	No
PLEKHG1	5034	-0.054	No
CEP78	5175	-0.058	No
MTA3	5189	-0.057	No
INPP4A	5287	-0.060	No
ARHGAP29	5378	-0.062	No
PALLD	5420	-0.062	No
RHBDD1	5450	-0.062	No
LRRN3	5481	-0.062	No
NINL	5490	-0.061	No
CR2	5603	-0.064	No
RAPGEF5	5719	-0.068	No
OXNAD1	5783	-0.070	No
EFNB2	5905	-0.074	No
ITGA6	6036	-0.078	No
TBCD	6251	-0.087	No
RDH10	6597	-0.101	No
JPH1	6862	-0.113	No
ZC3H6	6869	-0.112	No
SLFN5	6938	-0.115	No
ID1	6951	-0.115	No
CRIP1	7203	-0.126	No
FHIT	7332	-0.131	No
FAIM3	7477	-0.138	No
PSRC1	7753	-0.150	No
BCL2L11	7796	-0.151	No
CHST2	8127	-0.166	No
YPEL1	8448	-0.179	No
SCN7A	8951	-0.201	No
AGBL3	9194	-0.210	No
STAG3	9339	-0.216	No
AHRR	9658	-0.229	No
NDFIP2	9800	-0.234	No
RCAN1	9909	-0.238	No
NDST3	10158	-0.247	No
NSG1	10250	-0.250	No

KIF9	1313	0.613	Yes
CD63	1317	0.618	Yes
FNDC3B	1320	0.623	Yes
FAM46A	1328	0.628	Yes
TNFAIP2	1332	0.633	Yes
SPRY2	1338	0.638	Yes
TFPI	1404	0.640	Yes
MPL	1418	0.645	Yes
MECOM	1498	0.646	Yes
KLF11	1547	0.648	Yes
CDC42BPA	1691	0.647	Yes
GRB10	1809	0.646	Yes
LATS2	1823	0.650	Yes
TNS3	1912	0.650	Yes
SHANK3	1915	0.654	Yes
MN1	2041	0.653	Yes
MCTP2	2067	0.656	Yes
MAP3K8	2115	0.657	Yes
MYO5C	2194	0.658	Yes
SNTB1	2261	0.659	Yes
NFIL3	2317	0.660	Yes
FXYD5	2391	0.660	Yes
CPXM1	2433	0.662	Yes
DUSP6	2633	0.657	Yes
CRIM1	2660	0.659	Yes
NFKBIZ	2740	0.659	Yes
RPS11	2789	0.660	Yes
GYPC	2795	0.663	Yes
FOSL2	2804	0.666	Yes
LAPTM4B	2869	0.666	Yes
GNG11	2870	0.669	Yes
MYLK	2886	0.672	Yes
TNFRSF10B	3083	0.666	Yes
BATF	3145	0.666	Yes
SDPR	3180	0.668	Yes
UCHL1	3306	0.665	Yes
SOCS2	3468	0.660	Yes
HSH2D	3496	0.662	Yes
CPA3	3506	0.664	Yes
FHL1	3516	0.666	Yes
CD69	3519	0.669	Yes
TPM1	3521	0.671	Yes
CAPN2	3524	0.674	Yes
BHLHE40	3525	0.676	Yes
DUSP5	3586	0.676	No
EFHC2	3800	0.669	No
NLRP3	3826	0.670	No
MATR3	3861	0.671	No
BASP1	3921	0.671	No
ATP8B4	4068	0.666	No
NUDT11	4182	0.663	No
SLC22A15	4250	0.662	No
MBOAT7	4381	0.658	No
DRAM1	4441	0.658	No
SLC27A2	4640	0.651	No
FAM171B	4755	0.647	No
PDGFC	5119	0.633	No
RIPK2	5382	0.622	No
FAH	5468	0.620	No
CCND2	5470	0.621	No
EGR1	5727	0.611	No
TMEM220	5735	0.611	No
SLC39A8	5887	0.605	No
HOXB2	5890	0.606	No
ACY3	5923	0.606	No
CEBPD	6050	0.601	No
TUSC1	6088	0.600	No
CYB561	6104	0.600	No
STOM	6658	0.576	No
STAP1	6893	0.566	No
KCNQ5	7180	0.554	No
HOPX	7376	0.545	No

POU2AF1	10391	-0.255	No
APBA2	10449	-0.256	No
GAL3ST4	10611	-0.262	No
GAS7	10758	-0.267	No
CX3CR1	10866	-0.271	No
S1PR1	11101	-0.279	No
ADAMTS1	11356	-0.289	No
EVL	11375	-0.288	No
IKZF2	11638	-0.298	No
TSHR	11754	-0.302	No
PCSK5	11874	-0.305	No
SLC7A3	12006	-0.309	No
NETO2	12466	-0.328	No
TMOD2	12502	-0.328	No
GLRB	12787	-0.338	No
SUV420H1	12849	-0.339	No
PLCL1	13625	-0.371	No
NEIL3	13626	-0.369	No
SYNM	13747	-0.372	No
WT1	14113	-0.386	No
MBD5	14171	-0.387	No
ID2	14366	-0.393	No
PCDH9	14484	-0.396	No
ATAD2	14940	-0.414	No
SCN3A	15277	-0.426	No
PCDH10	15373	-0.428	No
DLEU2	15620	-0.436	No
HHIP	15683	-0.436	No
MME	15763	-0.437	No
NAPEPLD	15833	-0.438	No
CDH2	15903	-0.438	No
CYP2U1	15979	-0.439	No
FANCA	16117	-0.442	No
VCAN	16153	-0.441	No
PBK	16185	-0.440	No
IL7R	16226	-0.439	No
FXYD2	16538	-0.450	No
IPCEF1	16750	-0.457	No
AEBP1	16869	-0.459	No
SLC4A4	16978	-0.461	No
POLQ	17262	-0.471	No
HOMER1	17373	-0.472	No
ASPM	17379	-0.470	No
PTPRK	17490	-0.472	No
PPP1R1C	17543	-0.471	No
RHOU	17688	-0.474	No
AQP3	17833	-0.478	No
BIK	17936	-0.479	No
NKD2	17946	-0.476	No
LRRC1	17995	-0.476	No
MAL	18144	-0.479	No
MYBL1	18292	-0.482	No
FBXO43	18398	-0.484	No
GALNT2	18506	-0.485	No
NEGR1	18581	-0.485	No
LRRC28	18595	-0.482	No
P2RX5	18891	-0.492	No
SMPD3	18946	-0.491	No
MGAT4A	19097	-0.494	No
CCNG2	19220	-0.496	No
CCR8	19280	-0.495	No
ESCO2	19389	-0.497	No
RBMS3	19530	-0.499	No
BACH2	19576	-0.498	No
NEDD9	19624	-0.496	No
DNTT	19641	-0.493	No
CDKN2C	19717	-0.493	No
CDC25C	19833	-0.494	No
ZBTB1	19978	-0.497	No
PDE4D	20213	-0.503	Yes
EPM2AIP1	20231	-0.500	Yes
TANC1	20370	-0.502	Yes

ANKRD33B	7426	0.543	No
BSPRY	7520	0.539	No
MTHFD1L	7705	0.531	No
PHACTR1	7887	0.523	No
LDLRAD3	7926	0.522	No
B3GNT7	8251	0.508	No
LAMC1	8390	0.502	No
SPRY1	8581	0.494	No
SLC22A4	8690	0.490	No
RAB13	8804	0.486	No
TNFRSF1B	9005	0.477	No
SPARC	9169	0.471	No
RAMP1	9170	0.471	No
XBP1	9206	0.470	No
MYLIP	9290	0.467	No
CHRM3	9431	0.462	No
CD300LF	9453	0.462	No
CMBL	9680	0.452	No
TNFSF13B	9730	0.451	No
NIPAL2	9768	0.450	No
IL1B	9901	0.445	No
ZBTB8A	10028	0.441	No
SORL1	10148	0.436	No
IGFBP7	10154	0.437	No
LAPTM5	10199	0.436	No
SLC35F2	10335	0.431	No
SERPINE2	10478	0.425	No
C1QTNF4	11047	0.402	No
SLC7A5	11283	0.392	No
S100Z	11421	0.387	No
PVRL2	11712	0.376	No
NEK3	12156	0.358	No
PRSS2	12402	0.348	No
STON2	12861	0.329	No
SPINK2	13304	0.311	No
ADM	13365	0.310	No
CXCR7	13385	0.311	No
PROM1	13415	0.311	No
CLEC11A	13474	0.310	No
ADAM28	13623	0.305	No
ASB9	13845	0.297	No
CFD	14062	0.289	No
TTC7B	14669	0.264	No
CRYGD	14848	0.258	No
PTPRD	15343	0.238	No
SLC22A16	15517	0.232	No
BAALC	17146	0.163	No
NRIP3	17299	0.158	No
MGLL	17390	0.157	No
TRH	17667	0.147	No
KBTBD11	18498	0.113	No
CYTL1	18655	0.108	No
PDE4DIP	18952	0.098	No
F2RL1	19071	0.095	No
PROK2	19095	0.097	No
KCTD15	19117	0.098	No
HTR1F	19175	0.098	No
SPON1	19237	0.098	No
TIMP1	19830	0.075	No
SLC2A5	20039	0.069	No
PRAM1	20423	0.055	No
SYNGR1	20540	0.053	No
DPPA4	20676	0.050	No
ME3	20966	0.041	No
EPCAM	20977	0.043	No
GPR27	21492	0.025	No
TCTEX1D1	21586	0.024	No
PHTF1	21786	0.020	No
BCAT1	22231	0.005	No
SH3RF1	22848	-0.014	No
DUSP10	22869	-0.007	No
GBP4	22905	0.002	No

WDR7	20405	-0.499	Yes
FAM63B	20481	-0.499	Yes
IRS1	20511	-0.496	Yes
TCFL5	20559	-0.494	Yes
NOTCH3	20582	-0.491	Yes
RIMS3	20758	-0.494	Yes
COX6A1	20841	-0.493	Yes
HDAC4	20879	-0.491	Yes
SEC31B	21056	-0.494	Yes
APOLD1	21084	-0.490	Yes
CDC25B	21224	-0.492	Yes
GAS2	21363	-0.493	Yes
ARHGAP19	21464	-0.493	Yes
NCALD	21545	-0.491	Yes
HS3ST3B1	21595	-0.488	Yes
ITGAL	21773	-0.490	Yes
PTCRA	21814	-0.487	Yes
SEPT9	21820	-0.481	Yes
LYST	21843	-0.477	Yes
MNS1	21880	-0.473	Yes
PRKCA	21923	-0.469	Yes
SLC16A10	21982	-0.465	Yes
SPRED2	22073	-0.463	Yes
LBH	22103	-0.459	Yes
TUBB2A	22278	-0.460	Yes
RAG2	22294	-0.454	Yes
CD2	22351	-0.449	Yes
ADA	22414	-0.445	Yes
E2F2	22506	-0.441	Yes
ARPP21	22519	-0.434	Yes
SBK1	22543	-0.427	Yes
PLCG1	22551	-0.420	Yes
CD96	22585	-0.413	Yes
TNRC6C	22588	-0.405	Yes
SELPLG	22617	-0.398	Yes
CCR9	22628	-0.390	Yes
UBASH3A	22639	-0.383	Yes
CD8A	22641	-0.374	Yes
TRERF1	22661	-0.367	Yes
SIPA1L2	22711	-0.360	Yes
DGKA	22740	-0.352	Yes
ID3	22806	-0.345	Yes
CD28	22808	-0.335	Yes
SATB1	22813	-0.325	Yes
SH2D1A	22825	-0.316	Yes
LIG4	22828	-0.305	Yes
SH3KBP1	22829	-0.295	Yes
HIVEP3	22835	-0.285	Yes
IKZF3	22863	-0.275	Yes
CAMK4	22868	-0.264	Yes
GRAP2	22873	-0.253	Yes
THEMIS	22876	-0.241	Yes
GATA3	22878	-0.230	Yes
ITK	22887	-0.218	Yes
RASGRP1	22899	-0.206	Yes
FYB	22904	-0.193	Yes
TCF7	22907	-0.180	Yes
IGF2R	22910	-0.166	Yes
LCK	22911	-0.153	Yes
ITPR2	22915	-0.139	Yes
ETS1	22918	-0.125	Yes
CD247	22923	-0.110	Yes
ZAP70	22928	-0.095	Yes
BCL11B	22932	-0.080	Yes
LEF1	22933	-0.064	Yes
LAT	22938	-0.046	Yes
CD3E	22939	-0.026	Yes
CD3G	22941	0.000	Yes

Table S3: Gene Symbols in Gene Clusters I-V from Figure 2B

Gene Cluster I	Gene Cluster II	Gene Cluster III	Gene Cluster IV	Gene Cluster V
SNTB1	CRIM1	KATNBL1	RCAN1	IPP
DRAM1	CYTL1	OTTHUMG00000176181///RP11-119F7.5	PAFAH2	SLC16A10
IL1RAP	TNS3	GRSF1	SPRED2	STAG3
SDPR	PROM1	STAU2	GAS2	GSDMB
ARHGAP29	BAALC	TXLNG2P	CX3CR1	CD8A
HLX	LMO2	POU2AF1	NLGN4X	CD8B///LOC100996919
MEIS1	LAPTM4B	GATA3	NINL	LYST
NFIL3	BASP1	BATF	SMPD3	IRS1
KIT	FNDC3B	SPON1	SH3TC1	AQP3
IRAK3	FAM46A	DUSP6	CD1E	NCALD
TFPI	CREG1	SCN3A	CD1A	GAL3ST4
SLC22A4	BSPRY	HSPA1A///HSPA1B	CD1B	CCR9
CPA3	HOXA5	ADAMTS1	CD1D	CTC1
RIPK2	FLT3	CDH2	CD1C	PTPRK
PVRL2	RAB13	NDST3	AEBP1	SATB1
BHLHE40	CD302///LY75-CD302	RIMS3	E2F2	CD79A
PHACTR1	IL1B	TBCD	GTF2H2B	SEC31B
KLF9	EFHC2	APBA2	DOCK9	PARP8
FOSL2	SLC35F2	NSG1	DSERG1	TLR5
ARHGEF40	MCTP2	CR2	CDKN2D	PLCG1
CEBPD	ADAM28	SLC4A7	CD72	IKZF2
HBD	PHTF1	ZBTB1	PCDH9	IKZF3
CHRM3	IL18	WDR7	EFNB2	INPP4A
RAMP1	GNG11	NMT2	CYP2U1	ETS1
CRYGD	MN1	SUV420H1	GPR27	CD28
SYNGR1	HMGA2	WBP1L	MAML3	IL10RA
WHAMMP2///WHAMMP3	PDGFC	KIF3A	FRMD4A	PLCL1
MECOM	SLC27A2	SELPLG	RPS11	GRK5
CDC42BPA	KLF11	NREP	MAP1A	CRIP1
CFD	ATP8B4	UGP2	CCR8	NETO2
C11orf21	MAP3K8	MATR3///SNHG4	HIVEP3	MBD5
CD69	SLC25A13	HDAC4	BCL2L11	AGBL3
TUBB2A	ARMCX1	SRPK2	SLC16A6	PRKCA
S1PR1	PAM	EPM2AIP1	BIK	TMOD2
SERPINE2	F2RL1	ITPR2	DUSP5	CD3E
ELK3	GSAP	ZNF280D	DUSP3	DGKA
PDE4D	DPPA4	ITGA6	SCN7A	TRBC1
EGR1	CXCR7	CCNG2	HOXA3	GRAP2
ID2	NAP1L3	FANCA	RAB3D	FAM63B
MYLIP	GATM	GOLGA2P5	MYCN	FYB
SPRY2	ASB9	ARPP21	TNFRSF10B	LEF1
LAPTM5	PLA2G4A	POLQ	ICA1	SEPT9
CD44	IQCJ-SCHIP1///SCHIP1	STS	NRIP3	LIG4
TNFRSF1B	RAB31	CDKN2C	MOB3B	SYNE2
KBTBD11	NUDT11	PSRC1	ARSD	TCF7
HOXB2	XBP1	DLEU2	PDE4DIP	SH2D1A
SEMA4C	EPCAM	NEIL3	UCHL1	LCK
CCND2	SLC39A8	ASPM	VCAN	PVRIG
FXYD5	MEST	PBK	ADM	BCL11B
GYPC	CD63	ATAD2	HS3ST3B1	EVL
FHIT	SPRY1	APOLD1	ALOX5	CD96
DUSP10	STOM	PTCRA	RBMS3	RASGRP1
CAPN2	SORL1	TSHR	KIF13A	IL7R
HOPX	SERPINB1	RAPGEF5	KCTD15	CD3G
TIMP1	SPINK2	KIAA0226L	IGHG1	ITK
GRB10	MYO1F	RAG2	CDC25C	CHST2
CKAP4	LYL1	RAG1	NIPAL2	CD2
CTNNA1	CD34	NOTCH3	GLRB	ZAP70
TPM1	MPO	LRRC1		CD247
	SERPINB6	IGF2R		IL32
	BTK	KIAA0922		PCSK5
	CLDN10	LOC79015		HOMER1
	MYO5C	MAL		NEDD9
	HPGDS	ARHGAP19		CHI3L2
	MGLL	MYBL1		ITGAL
	NFE2	COX6A1		MGAT4A
	FAH	CDC25B		LAT

ANGPT1	YPEL1	LBH
SLC2A5	MME	ID3
PYGL	AKAP2///PALM2-AKAP2	FAM69A
CTBP2	GALNT2	CAMK4
VCL	LDLRAD4	UBASH3A
IGFBP7	CEP70	ATM
SPARC	DNTT	IPCEF1
DEPTOR	ADA	BACH2
KIAA0125	MNS1	FAIM3
PRSS2///PRSS3	TFDP2	LRRN3
MYLK	FXYD2	TRAF3IP3
PRSS2	SLC4A4	P2RX5
LILRA2	RUFY3	GBP1
LAMC1	GAS7	
HTR1F	HNRNPR	
MPL	IGHM	
NLRP3	PALLD	
MBOAT7	SERHL2	
CYB561		
TRH		
WT1		
GSTM3		
SYNM		
LAT2		
PTPRD		
MAP7		
P2RY2		
ERG		
CD33		
GOLIM4		
IRF5		
TNFAIP2		
EIF4EBP1		
ID1		
FHL1		
BCAT1		
SLC7A5		
FAM49A		
TCF4		
PLEK		
PLXNB2		
LYN		
MEF2C		
ALCAM		
HHEX		
STAP1		
NEK3		
ME3		
SOCS2		
CLEC11A		
